# A Multifaceted Intervention to Implement Guidelines and Improve Admission Paediatric Care in Kenyan District Hospitals: A Cluster Randomised Trial

**DOI:** 10.1371/journal.pmed.1001018

**Published:** 2011-04-05

**Authors:** Philip Ayieko, Stephen Ntoburi, John Wagai, Charles Opondo, Newton Opiyo, Santau Migiro, Annah Wamae, Wycliffe Mogoa, Fred Were, Aggrey Wasunna, Greg Fegan, Grace Irimu, Mike English

**Affiliations:** 1 KEMRI-Wellcome Trust Research Programme, Nairobi, Kenya; 2 Division of Child Health, Ministry of Public Health and Sanitation, Nairobi, Kenya; 3 Ministry of Medical Services, Nairobi, Kenya; 4 Department of Paediatrics and Child Health, University of Nairobi, Kenyatta National Hospital, Nairobi, Kenya; 5 Infectious Disease Epidemiology Unit, Department of Epidemiology and Population Health, London School of Hygiene and Tropical Medicine, London, United Kingdom; 6 Department of Paediatrics, University of Oxford and John Radcliffe Hospital, Headington, Oxford, United Kingdom; University of Edinburgh and Andrija Stampar School of Public Health, Scotland

## Abstract

**Background:**

In developing countries referral of severely ill children from primary care to district hospitals is common, but hospital care is often of poor quality. However, strategies to change multiple paediatric care practices in rural hospitals have rarely been evaluated.

**Methods and Findings:**

This cluster randomized trial was conducted in eight rural Kenyan district hospitals, four of which were randomly assigned to a full intervention aimed at improving quality of clinical care (evidence-based guidelines, training, job aides, local facilitation, supervision, and face-to-face feedback; *n* = 4) and the remaining four to control intervention (guidelines, didactic training, job aides, and written feedback; *n* = 4). Prespecified structure, process, and outcome indicators were measured at baseline and during three and five 6-monthly surveys in control and intervention hospitals, respectively. Primary outcomes were process of care measures, assessed at 18 months postbaseline.

In both groups performance improved from baseline. Completion of admission assessment tasks was higher in intervention sites at 18 months (mean = 0.94 versus 0.65, adjusted difference 0.54 [95% confidence interval 0.05–0.29]). Uptake of guideline recommended therapeutic practices was also higher within intervention hospitals: adoption of once daily gentamicin (89.2% versus 74.4%; 17.1% [8.04%–26.1%]); loading dose quinine (91.9% versus 66.7%, 26.3% [−3.66% to 56.3%]); and adequate prescriptions of intravenous fluids for severe dehydration (67.2% versus 40.6%; 29.9% [10.9%–48.9%]). The proportion of children receiving inappropriate doses of drugs in intervention hospitals was lower (quinine dose >40 mg/kg/day; 1.0% versus 7.5%; −6.5% [−12.9% to 0.20%]), and inadequate gentamicin dose (2.2% versus 9.0%; −6.8% [−11.9% to −1.6%]).

**Conclusions:**

Specific efforts are needed to improve hospital care in developing countries. A full, multifaceted intervention was associated with greater changes in practice spanning multiple, high mortality conditions in rural Kenyan hospitals than a partial intervention, providing one model for bridging the evidence to practice gap and improving admission care in similar settings.

**Trial registration:**

Current Controlled Trials ISRCTN42996612

*Please see later in the article for the Editors' Summary*

## Abstract 2

Philip Ayieko and colleagues report the outcomes of a cluster-randomized trial carried out in eight Kenyan district hospitals evaluating the effects of a complex intervention involving improved training and supervision for clinicians. They found a higher performance of hospitals assigned to the complex intervention on a variety of process of care measures, as compared to those receiving the control intervention.

## Editors' Summary

**Background:**

In 2008, nearly 10 million children died in early childhood. Nearly all these deaths were in low- and middle-income countries—half were in Africa. In Kenya, for example, 74 out every 1,000 children born died before they reached their fifth birthday. About half of all childhood (pediatric) deaths in developing countries are caused by pneumonia, diarrhea, and malaria. Deaths from these common diseases could be prevented if all sick children had access to quality health care in the community (“primary” health care provided by health centers, pharmacists, family doctors, and traditional healers) and in district hospitals (“secondary” health care). Unfortunately, primary health care facilities in developing countries often lack essential diagnostic capabilities and drugs, and pediatric hospital care is frequently inadequate with many deaths occurring soon after admission. Consequently, in 1996, as part of global efforts to reduce childhood illnesses and deaths, the World Health Organization (WHO) and the United Nations Children's Fund (UNICEF) introduced the Integrated Management of Childhood Illnesses (IMCI) strategy. This approach to child health focuses on the well-being of the whole child and aims to improve the case management skills of health care staff at all levels, health systems, and family and community health practices.

**Why Was This Study Done?:**

The implementation of IMCI has been evaluated at the primary health care level, but its implementation in district hospitals has not been evaluated. So, for example, interventions designed to encourage the routine use of WHO disease-specific guidelines in rural pediatric hospitals have not been tested. In this cluster randomized trial, the researchers develop and test a multifaceted intervention designed to improve the implementation of treatment guidelines and admission pediatric care in district hospitals in Kenya. In a cluster randomized trial, groups of patients rather than individual patients are randomly assigned to receive alternative interventions and the outcomes in different “clusters” of patients are compared. In this trial, each cluster is a district hospital.

**What Did the Researchers Do and Find?:**

The researchers randomly assigned eight Kenyan district hospitals to the “full” or “control” intervention, interventions that differed in intensity but that both included more strategies to promote implementation of best practice than are usually applied in Kenyan rural hospitals. The full intervention included provision of clinical practice guidelines and training in their use, six-monthly survey-based hospital assessments followed by face-to-face feedback of survey findings, 5.5 days training for health care workers, provision of job aids such as structured pediatric admission records, external supervision, and the identification of a local facilitator to promote guideline use and to provide on-site problem solving. The control intervention included the provision of clinical practice guidelines (without training in their use) and job aids, six-monthly surveys with written feedback, and a 1.5-day lecture-based seminar to explain the guidelines. The researchers compared the implementation of various processes of care (activities of patients and doctors undertaken to ensure delivery of care) in the intervention and control hospitals at baseline and 18 months later. The performance of both groups of hospitals improved during the trial but more markedly in the intervention hospitals than in the control hospitals. At 18 months, the completion of admission assessment tasks and the uptake of guideline-recommended clinical practices were both higher in the intervention hospitals than in the control hospitals. Moreover, a lower proportion of children received inappropriate doses of drugs such as quinine for malaria in the intervention hospitals than in the control hospitals.

**What Do These Findings Mean?:**

These findings show that specific efforts are needed to improve pediatric care in rural Kenya and suggest that interventions that include more approaches to changing clinical practice may be more effective than interventions that include fewer approaches. These findings are limited by certain aspects of the trial design, such as the small number of participating hospitals, and may not be generalizable to other hospitals in Kenya or to hospitals in other developing countries. Thus, although these findings seem to suggest that efforts to implement and scale up improved secondary pediatric health care will need to include more than the production and dissemination of printed materials, further research including trials or evaluation of test programs are necessary before widespread adoption of any multifaceted approach (which will need to be tailored to local conditions and available resources) can be contemplated.

**Additional Information:**

Please access these Web sites via the online version of this summary at http://dx.doi.org/10.1371/journal.pmed.1001018.

- WHO provides information on efforts to reduce global child mortality and on Integrated Management of Childhood Illness (IMCI); the WHO pocket book “Hospital care for children contains guidelines for the management of common illnesses with limited resources (available in several languages)
- UNICEF also provides information on efforts to reduce child mortality and detailed statistics on child mortality
- The iDOC Africa Web site, which is dedicated to improving the delivery of hospital care for children and newborns in Africa, provides links to the clinical guidelines and other resources used in this study

## Introduction

Common illnesses including pneumonia, malaria, and diarrhea remain major contributors to child mortality in low-income countries [1]. Hospital care of severe illnesses may help improve survival, and disease-specific clinical guidelines have been provided by the World Health Organization (WHO) for more than 15 y [2], and as collated texts since 2000 [3],[4]. These guidelines form part of the Integrated Management of Childhood Illnesses (IMCI) approach adopted by over 100 countries. However, in contrast to its primary care aspects [5],[6], implementation of IMCI at district hospitals has not been evaluated. Paediatric hospital care is often inadequate in our setting and also in other low-income countries both in Africa and Asia [7]–[10], with most inpatient deaths occurring within 48 h of admission [11].

We therefore set out to develop and test a strategy to improve paediatric care in district hospitals in partnership with the Kenyan government [12]–[14]. We considered a trial of alternative interventions necessary for ethical reasons and because systematic reviews indicated uncertainty in the value of multicomponent interventions [15]. Our evaluation is based on the classical Donabedian approach—assessing structure, process, and valued health system outcome measures [16]. We randomised hospitals, rather than individuals, to intervention groups because the intervention was designed to influence how the paediatric teams provided care. Secondly, the cluster randomised trial offered logistical convenience in implementing certain intervention components, which by their nature (training, feedback, supervision) are easier to administer to groups rather than on an individual basis. To provide data to inform debate on the plausibility of any cause–effect relationship arising from the trial data, we also planned that evaluation spanned a realistic timescale, evaluated possible postintervention deterioration, and assessed intervention context, adequacy, and barriers to implementation [12],[17]–[20].

## Methods

### Study Sites and Participants

Eight rural hospitals (H1 to H8) were chosen purposefully from four of Kenya's eight provinces to provide some representation of the variety of rural hospital settings encountered in Kenya (Table 1) [12]. Hospitals admitting a minimum of 1,000 children and conducting at least 1,200 deliveries per year were eligible for inclusion. Prior to the study, medical records documenting admission information were written as nonstandard, free-text notes in all eight hospitals. The Ministry of Health usually aims to disseminate national guidelines aimed at hospital care to facilities through distribution of some print materials and ad hoc or opportunistic workshops or seminars. It had not previously been able to augment this approach with systematic efforts or provide specific supervision to support paediatric hospital care. Further, none of the eight hospitals themselves had explicit procedures for implementing new clinical guidelines.

**Table 1:** Baseline hospital characteristics and characteristics of 8,205 paediatric admission events at baseline and during the 18-mo intervention period.

Characteristic	H1^a^	H2^a^	H3^a^	H4^a^	H5^a^	H6^a^	H7^a^	H8^a^
*Hospital characteristics*								
Malaria transmission setting	Intense	Moderate	Highland	Arid	Intense	Arid	Highland	Moderate
Antenatal HIV prevalence	High	Low	High	Moderate	High	Moderate	High	Low
Infant mortality rate (per 1,000)	>100	∼40	∼70	∼70	>100	∼70	>100	∼40
Catchment population with income below US$2 per day (%)	50–70	∼35	50–70	50–70	50–70	50–70	50–70	∼35
Annual paediatric admissions	2,356	3,160	4,205	996	2,925	1,058	4,738	2,128
All cause paediatric ward mortality rate	13.7%	5.40%	7.30%	8.00%	6.50%	5.20%	4.10%	7.30%
Consultant specialists [pediatricians]	3 [0]	3 [0]	5 [1]	2 [0]	2 [0]	1 [0]	5 [1]	2 [0]
General medical officers	2	4	5	4	4	4	5	4
Clinical officers	15	21	29	19	18	18	27	20
Nurses in whole hospital (all cadres)	140	161	207	120	114	128	284	144
Doctors and clinical officers at initial training	9	10	14	6	12	2	8	5
Nurses at initial training	24	20	19	23	24	26	25	32
Number of trained staff/*n* targeted for training	33/32^b^	31/32	35/32^b^	29/32	37/40	35/40	43/40^b^	42/40^b^
Medical staff responsible for admissions at survey 4 and trained, *n* (%)	3 (13)	3 (10)	2 (6)	1 (5)	6 (26)	1 (6)	0 (0)	1 (4)
*Characteristics of patients enrolled during survey 1 to survey 4*								
Admission episodes at baseline^c^	246	330	261	293	121	285	276	323
Admission episodes at 18 mo^c^	230	312	309	307	271	198	341	337
Admission episodes included in descriptive analyses (survey 1, 2, 3, and 4)	948	1,308	1,089	1,039	638	908	1,128	1,147
Age in months, mean (SD)	16.1 (12.8)	16.7 (13.1)	16.2 (12.5)	17.1 (13.6)	16.9 (13.1)	15.2 (12.9)	17.5 (13.3)	14.7 (12.4)
Male, *n* (%)	448 (53.2)	570 (57.7)	470 (55.6)	421 (56.5)	332 (54.2)	422 (55.6)	498 (52.7)	544 (54.3)
All cause paediatric ward mortality rate (survey 1)	14/246 (5.7)	12/330 (3.6)	5/261 (1.9)	33/293 (11.3)	3/121 (2.5)	20/285 (7.0)	4/276 (1.5)	18/323 (5.6)
*Clinical diagnoses* d, *n (%)*								
Diarrhoea/dehydration	271 (28.6)	290 (22.2)	312 (28.7)	257 (24.7)	89 (13.9)	219 (24.1)	210 (18.6)	381 (33.2)
Malaria	758 (80.0)	866 (66.2)	750 (68.9)	799 (76.9)	458 (71.8)	572 (63.0)	920 (81.6)	479 (41.8)
Pneumonia	407 (42.9)	819 (62.6)	469 (43.1)	547 (52.6)	158 (24.8)	499 (55.0)	423 (37.5)	573 (50.0)

a H1–H4 are intervention hospitals; H5–H8 are control hospitals.

b Number of staff trained exceeded target.

c Survey data correspond to records retrieved on randomly sampled calendar dates in the 6-mo period prior to the survey.

d Denominator is all admission events from surveys 1–4, number of diagnoses are episodes and therefore can be greater than number of admissions.

We collected data from medical records of paediatric admissions aged 2–59 mo to describe paediatric care practices of clinicians and nursing staff targeted by the guidelines, training, and feedback. The Kenya Medical Research Institute National Ethics and Scientific review committees approved the study (Texts S1 and S2).

### Randomization and Masking

Prior to inclusion in the study the eight shortlisted hospitals were visited and meetings were held with the hospital management team. At these meetings, the study design, randomization, potential inputs, approach to data collection, and longevity were explained. All hospital management teams subsequently assented to their hospital's participation and randomization after internal discussions. Assent from the hospital's catchment population was not sought. Staff in all hospitals were made aware of the study's overall aims to explore ways to improve care and need for data collection through specific presentations made after randomization at the start of introductory training and using written information sheets. After obtaining the hospitals' assent we allocated eight hospitals (clusters) to a full (intervention group, hospitals H1–H4) or partial (control group, hospitals H5–H8) package of interventions using restricted randomization. Of 70 possible allocations, seven defined two relatively balanced groups (Table 1). These allocations were written on identical pieces of paper, with hospitals represented by codes, and one allocation was randomly selected using a “blind draw” procedure. Participating hospitals and the research team could not be masked to group allocation. However, information on group allocation was not publicly disseminated and the geographic distance between hospitals was large. We therefore do not feel that users of the hospitals were aware of or influenced by the form of intervention allocated to the hospital.

### Study Intervention

**Figure 1:**
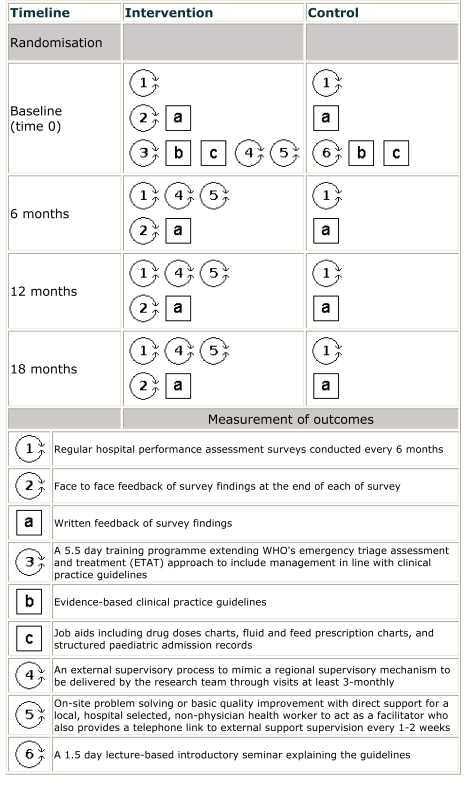
Graphical depiction of the complex intervention delivered over an 18-mo period (adapted from Perera et al. [**21**]). Circles represent activities and squares represent objects; components delivered concurrently appear side by side.

The intervention delivered over 18 mo (from September 2006 to April 2008) aimed to improve paediatric admission care by promoting hospitals' implementation of best-practice guidelines and local efforts to tackle local organizational constraints. Before the trial commenced, a decision was made to adjust the timing of the primary endpoint for measuring intervention effectiveness, aligning it with the end of this 18-mo active intervention period. As part of this updated approach, monitoring of intervention sites was planned to continue for 12 mo after active intervention had ended. Funds were not available to support comparable extended monitoring in control sites. The intervention components are labeled 1–6 and a–c in Figure 1 [21] and included: (1) setting up a scheme for regular hospital assessment through surveys conducted six monthly, followed by (2) face-to-face feedback of findings in intervention sites, and (a) written feedback in both groups. The other components were: (3) 5.5-d training aimed at 32 health workers of all cadres approximately 6–10 wk after baseline surveys (July to August 2006) in intervention hospitals [13], (b) provision of clinical practice guidelines introduced with training, (c) job aides, (4) an external supervisory process, and (5) identification of a full-time local facilitator (a nurse or diploma-level clinician) responsible for promoting guideline use and on-site problem solving [19]. Supervision visits were approximately two to three monthly, but facilitation remained in place throughout the 18 mo. The package for control sites (H5–H8) included five components (1, 6, a, b, and c): (1) six-monthly surveys with written feedback only, provision of (b) clinical practice guidelines and (c) job aides, and (6) a 1.5-d initial guideline seminar for approximately 40 hospital staff. The design thus compares two alternative intensities of intervention, both providing considerably more than routinely delivered, although we refer to one arm as the “control.”

One of the job aides, introduced to all sites with all training and continuously supplied to improve documentation of illness, was a paediatric admission record (PAR) form. This was to replace traditional “blank paper” medical notes [22]. All hospitals were aware that their records and patient management were to be regularly evaluated. All job aides, training materials, and assessment tools are available online (http://www.idoc-africa.org/docs/list/cat/5/subcat/27).

### Data Collection

Data were collected at baseline and then at six-monthly intervals during six and four surveys in intervention (surveys 1–6) and control hospitals (surveys 1–4), respectively (Figure 1). A single survey took approximately 2 wk with all sites surveyed within a maximum 6-wk consecutive period by employing up to four teams. The survey tools and team training have been described in detail elsewhere [14]. In brief, data were collected using three tools adapted from previous work [7],[8] then extensively pretested: a checklist of structure indicators, patient case-record data abstraction forms, and a structured parent/guardian interview tool. In the case of the parent/guardian interview formal, written consent was obtained prior to data collection with no parent/guardians refusing consent. Ethical approval was granted for confidential abstraction of data from archived case records without individuals' consent. Survey team leaders remained the same throughout the study and teams received 3 wk initial training that included a pilot survey. Data collectors could not be blinded to allocation, but all were guided by standard operating procedures and, for case records, a 10% sample were independently reevaluated by the survey supervisor during each survey. Agreement rates for data abstracted were consistently greater than 95%.

Case records from a random sample of calendar dates from the 6-mo intersurvey periods were selected with the proportion of dates sampled adjusted to yield approximately 400 records based on hospitals' admission rates. On the basis of prior experience we aimed to conduct interviews with 50 caretakers of admitted children during each 2 wk survey (surveys 1–4).

### Performance Indicators

Primary effectiveness measures were 14 process indicators measured on paediatric admissions aged 2–59 mo at 18-mo post baseline (survey 4). Secondary measures were four valued system outcomes of admission and changes in structure measured at the hospital level. The trial was not designed to evaluate mortality effects.

#### Process indicators

Indicators reflected standards defined by the clinical guidelines focusing on: pneumonia, malaria and diarrhoea, and/or dehydration that account for more than 65% of paediatric admissions and deaths [13]. These span assessment, therapeutic, and supportive care. We defined dichotomous variables for process errors, e.g. wrong intravenous fluid prescription. However, to summarize assessment an aggregate assessment score for each child (range 0–1) was calculated by counting the number of features documented and dividing this by the total relevant for each child according to guidelines (pneumonia 8, malaria and diarrhoea/dehydration both 6). The denominator of the score was thus child specific, depended on the extent of comorbidity, and had a maximum value of 16 due to two shared features of severe illness.

#### Outcome indicators

These indicators reflected adherence to key policy recommendations and included vitamin A prescription, identifying missed opportunities for immunization, and universal provider initiated testing and counselling (PITC) for HIV. A fourth was based on a score (range 0–4) reflecting caretakers' correct knowledge, at discharge, of their child's diagnosis and number, duration, and frequency of discharge drugs.

#### Structure indicators

The availability of equipment, basic supplies, and service organization were evaluated using a checklist of 113 items needed to provide guideline directed care and representing seven logical groupings [23]. Data were collected by observation and interviewing senior hospital staff. A simple, unweighted proportion of the 113 items was derived, the change in proportion available from survey 1 to survey 4 was calculated for each hospital and the mean change in intervention and control groups compared.

### Sample Size

There were 70 district hospitals in Kenya at the time of the study. Hospitals from four of Kenya's eight provinces without potentially confounding, nonstudy interventions and meeting the outlined eligibility criteria were shortlisted. Data on additional criteria felt to help define the range of contexts in Kenya were then evaluated, and eight hospitals from four provinces were purposefully selected to ensure that at least two out of these eight hospitals met each positive and negative criterion (Table 1), that two hospitals were from each of the four provinces and represented logistical implications of their location. The sample size of eight hospitals was estimated using two approaches to compare performance within each hospital (plausibility design) and across the two arms of the trial (cluster RCT analysis). Within hospitals, we estimated that 50% correct performance could be estimated with precision (95% confidence intervals [CIs]) of ±7% with 200 admission records (50% of 400 sampled admissions), or, ±10% with 100 admission records. The second calculation for group (C-RCT) comparisons accounted for the clustered nature of the data. The median intraclass correlation coefficient (ICC) for 46 quality of care variables estimated from a health facility cluster survey in Benin was ρ = 0.2 [24]. We estimated, employing this value for the ICC, that 100 observations per cluster would provide 80% power to detect a 50% or greater difference in proportions between intervention and control arms at 18 mo follow-up [25].

### Statistical Analysis

Data were double entered and verified in Microsoft Access and analysed using Stata, version 10 (Stata Corp.) according to the prespecified analysis plan.

#### Descriptive analysis

We present characteristics of hospitals at baseline and of children contributing admission data during surveys 1–4. Process and outcome indicators are summarized as percentages and the absolute changes (95% CI) between survey 1 and 4 calculated for each hospital.

#### Comparison of intervention and control arms

Two approaches were used. The first approach was a cluster level analysis of mean change from baseline in intervention (*n* = 4) and control (*n* = 4) groups, a test of mean difference-in-difference, using an unpaired *t*-test (with individual sample variances if appropriate), which is reasonably robust to deviations from normality, even for a small number of clusters. The second approach compared the groups at survey 4 using a two-stage method [26]. In the initial stage, logistic or linear regression analyses were conducted for each outcome adjusting for hospital-level covariates (all-cause paediatric mortality, malaria transmission, and size) and gender, illness outcome (alive or died) at the patient-level but not study group. The observed events were then subtracted from predicted events in the regressions to obtain a residual for each cluster. The cluster residuals were then compared in the second stage using a *t*-test [26].

#### Performance post intervention period

Data from intervention hospitals (surveys 4–6) were analysed to determine the impact of intervention withdrawal by assessing trends graphically and using regression analysis. Linear and binomial regression analysis was used to assess whether the means or proportions changed over time; this was done by testing to see whether there was a linear trend associated with the postintervention period (surveys 4–6).

We acknowledge the use of multiple significance tests and report 95% CIs and exact *p*-values where appropriate noting that *p*-values lower than those traditionally considered “significant” might be given greater weight. We would, however, suggest consideration of the plausibility of the intervention's effectiveness should also take into account any consistency in effect across indicators.

## Results

**Figure 2:**
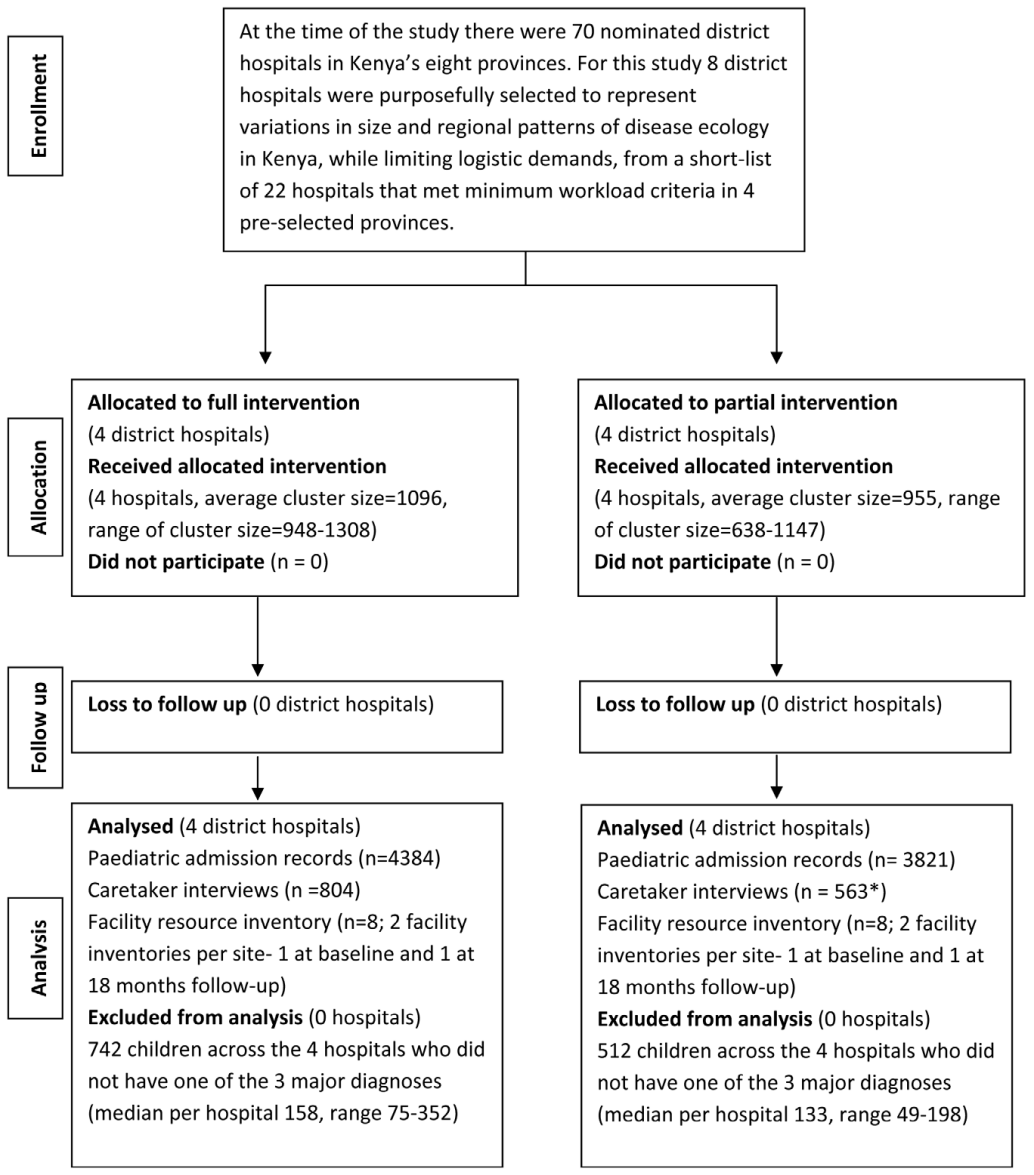
Trial profile. *Caretaker interviews not conducted in control sites 12 mo after intervention (see Tables S1, S2, S3, S4, S5).

All hospitals participated in each survey as planned (Figure 2). The intervention's implementation is summarized in Table 1 and showed that intended training for at least 32 workers (the majority were nurses) was attained in three of the four intervention sites. No hospital received additional, nonstudy paediatric training during the study period. Staff turnover, which was of a like-for-like nature, was high in both intervention and control hospitals, especially in the larger hospitals (H3 and H7). At 18 mo only, 5% (2/35) to 13% (3/23) and 0 to 26% (6/23) of frontline clinical staff in the intervention and control hospitals, respectively, had received initial training (Table 1). As part of supervisory activities, the implementation team conducted an additional 10–12-h training session in two intervention hospitals and two to three small group sessions of 2–4 h in all four intervention hospitals over the 18 mo intervention period.

Intervention and control sites were similar at baseline (Table 1), although routinely reported prior paediatric mortality varied from 4.1% to 13.4%. Case records for primary process of care indicators were available for 1,130 and 1,005 records at baseline and 1,158 and 1,157 case records at 18 mo for intervention and control hospitals, respectively (Table 1). Additional data summarizing the patient populations at cluster level are provided in Tables S1 and S2.

### Primary Effectiveness Measures

Results were similar from both approaches used to compare intervention arms, i.e., adjusted comparison at 18 mo and difference of differences. For brevity, we outline only the results of adjusted comparisons and present other data in Tables S3, S4, S5.

#### Process indicators

Of 14 process of care indicators, performance at hospital level for three indicators assessed for every admission were highly variable but often poor at baseline (Table 2): e.g., documentation of weight, 1%–95%, and mean assessment scores 0.26–0.44. In addition, disease-specific treatment practices at baseline were poor, rarely conforming to guideline recommendations (Table 2). For example, prescription of nationally recommended (since 1998) loading dose quinine for <7% appropriate cases in seven sites at baseline (Table 2).

**Table 2:** Changes in process and outcome indicators between baseline and 18 mo postintervention by hospital.

Process/Outcome Care Indicator	H1	H2	H3	H4	H5	H6	H7	H8
	*n* (Percent [95% CI])	*n* (Percent [95% CI])	*n* (Percent [95% CI])	*n* (Percent [95% CI])	*n* (Percent [95% CI])	*n* (Percent [95% CI])	*n* (Percent [95% CI])	*n* (Percent [95% CI])
Child's weight documented								
Survey 1	113/246 (46 [40–52])	103/330 (31 [26]–[37])	247/261 (95 [91–97])	192/293 (66 [60–71])	59/121 (48 [40–58])	9/285 (3 [1]–[6])	86/276 (31 [26]–[37])	3/323 (1 [0–3])
Survey 4	201/230 (87 [82–91])	304/312 (97 [95–99])	286/309 (93 [89–95])	186/307 (61 [55–66])	123/271 (45 [39–52])	163/198 (82 [76–87])	269/341 (79 [74–83])	156/337 (46 [41–52])
Percent difference (95% CI)	41% (34–49)	66% (61–72)	−2% (−6 to 2)	−5% (−13 to 3)	−3% (−14 to 7)	79% (74–84)	48% (41–55)	45% (40–51)
Child's temperature documented								
Survey 1	7/246 (3 [1]–[6])	37/330 (11 [8]–[15])	10/261 (4 [2]–[7])	87/293 (30 [25]–[35])	2/121 (2 [0–6])	251/285 (88 [84–92])	23/276 (8 [5]–[12])	7/323 (2 [1]–[4])
Survey 4	125/230 (54 [48–61])	280/312 (90 [86–93])	233/309 (75 [70–80])	209/307 (68 [63–73])	3/271 (1 [0–3])	187/198 (94 [90–97])	180/341 (53 [47–58])	128/337 (38 [33]–[43])
Percent difference (95% CI)	52% (45–58)	79% (74–83)	72% (66–77)	38% (31–46)	−1% (−3 to 2)	6% (1–12)	44% (38–51)	36% (30–41)
Average assessment score^a^								
Survey 1	0.30 (0.29–0.32)	0.30 (0.29–0.31)	0.34 (0.33–0.36)	0.31 (0.30–0.32)	0.26 (0.23–0.28)	0.32 (0.31–0.34)	0.44 (0.42 to −0.45)	0.26 (0.24 to −0.27)
Survey 4	0.88 (0.85–0.91)	0.97 (0.96–0.98)	0.97 (0.96–0.98)	0.92 (0.91–0.93)	0.38 (0.35–0.40)	0.72 (0.67–0.76)	0.65 (0.62–0.68)	0.84 (0.82–0.85)
Difference (95% CI)	0.58 (0.55–0.61)	0.67 (0.66–0.69)	0.62 (0.61–0.64)	0.61 (0.59–0.63)	0.12 (0.08–0.16)	0.39 (0.35–0.43)	0.21 (0.18–0.25)	0.58 (0.56–0.60)
Severe malaria episodes with twice daily quinine maintenance dose								
Survey 1	0/154 (0)	0/72 (0)	0/161 (0)	3/192 (2 [0–4])	3/94 (3 [1]–[9])	34/94 (36 [27]–[47])	1/234 (0)	0/88 (0)
Survey 4	117/152 (77 [69–83])	149/154 (97 [93–99])	72/77 (94 [85–98])	175/208 (84 [78–89])	43/76 (57 [45–68])	31/64 (48 [36–61])	35/81 (43 [32–55])	20/58 (34 [22–48])
Percent difference (95% CI)	77% (70–84)	97% (93–100)	94% (90–97)	83% (77–88)	53% (42–64)	12% (−3 to 28)	43% (36–49)	34% (24–45)
Severe malaria episodes with quinine loading								
Survey 1	6/162 (4 [1]–[8])	5/84 (6 [2]–[13])	3/169 (2 [0–5])	11/205 (5 [3]–[9])	7/104 (7 [3]–[13])	55/105 (52 [42–62])	0/236 (0)	0/125 (0)
Survey 4	149/168 (89 [83–93])	160/163 (98 [95–100])	86/88 (98 [92–100])	181/218 (83 [77–88])	112/116 (97 [91–99])	22/71 (31 [21]–[43])	84/89 (94 [87–98])	31/69 (45 [33–57])
Percent difference (95% CI)	85% (79–91)	92% (88–97)	96% (92–100)	78% (72–84)	90% (84–96)	−21% (−36 to −7)	94% (91–97)	45% (36–54)
Severe malaria episodes with quinine daily dose ≥40 mg/kg								
Survey 1	25/161 (16 [10]–[22])	4/79 (5 [1]–[12])	4/168 (2 [1]–[6])	13/205 (6 [3]–[11])	11/102 (11 [6]–[18])	14/101 (14 [8]–[22])	11/236 (5 [2]–[8])	34/125 (27 [20]–[36])
Survey 4	0/159 (0)	1/163 (1 [0–3])	1/85 (1 [0–6])	5/218 (2 [1]–[5])	6/84 (7 [3]–[15])	4/71 (6 [2]–[14])	1/89 (1 [0–6])	11/69 (16 [8]–[27])
Percent difference (95% CI)	−16% (−21 to −10)	−4% (−8 to −1)	−1% (−5 to 2)	−4% (−8 to 0)	−4% (−12 to 5)	−8% (−18 to 1)	−4% (−8 to 1)	−11% (−24 to 1)
Gentamicin prescriptions with once daily dose								
Survey 1	1/99 (1 [0–5])	2/191 (1 [0–4])	2/125 (2 [0–6])	5/133 (4 [1]–[9])	0/21 (0)	20/183 (11 [7]–[16])	1/51 (2 [0–10])	3/236 (1 [0–4])
Survey 4	62/75 (83 [72–90])	134/138 (97 [93–99])	41/46 (89 [76–96])	103/117 (88 [81–93])	68/90 (76 [65–84])	78/114 (68 [59–77])	72/84 (86 [76–92])	129/190 (68 [61–74])
Percent difference (95% CI)	82% (74–90)	96% (93–99)	88% (81–94)	84% (78–91)	76% (57–94)	57% (49–66)	84% (74–94)	67% (60–73)
Gentamicin prescriptions with daily dose <4 mg/kg								
Survey 1	46/99 (46 [36–57])	41/191 (21 [16]–[28])	21/125 (17 [11]–[25])	20/133 (15 [9]–[22])	13/21 (62 [38–82])	19/183 (10 [6]–[16])	7/51 (14 [6]–[26])	18/236 (8 [5]–[12])
Survey 4	1/75 (1 [0–7])	3/138 (2 [0–6])	0/46 (0)	6/117 (5 [2]–[11])	15/90 (17 [10]–[26])	8/114 (7 [3]–[13])	5/84 (6 [2]–[13])	12/190 (6 [3]–[11])
Percent difference (95% CI)	−45% (−57 to −33)	−19% (−26 to −12)	−17% (−28 to −6)	−10% (−17 to −2)	−45% (−64 to −26)	−3% (−10 to 3)	−8% (−18 to 2)	−1% (−6 to 4)
Gentamicin prescriptions with daily dose ≥10 mg/kg								
Survey 1	3/99 (3 [1]–[9])	1/191 (1 [0–3])	6/125 (5 [2]–[10])	9/133 (7 [3]–[12])	1/21 (5 [0–24])	13/183 (7 [4]–[12])	4/51 (8 [2]–[19])	21/236 (9 [6]–[13])
Survey 4	7/75 (9 [4]–[18])	2/138 (1 [0–5])	3/46 (7 [1]–[18])	9/117 (8 [4]–[14])	13/90 (14 [8]–[23])	7/114 (6 [3]–[12])	2/84 (2 [0–8])	31/190 (16 [11]–[22])
Percent difference (95% CI)	6% (−1 to 13)	1% (−1 to 3)	2% (−6 to 9)	1% (−6 to 7)	10% (−6 to 26)	−1% (−7 to 5)	−5% (−13 to 2)	7% (1 to 14)
Correct intravenous fluid prescription								
Survey 1	2/33 (6 [1]–[20])	2/16 (13 [2]–[38])	0/85 (0)	3/28 (11 [2]–[28])	0/9 (0)	3/25 (12 [3]–[31])	11/27 (41 [22–61])	1/14 (7 [0–34])
Survey 4	28/53 (53 [39–67])	57/69 (83 [72–91])	50/65 (77 [65–86])	39/69 (57 [44–68])	8/25 (32 [15–54])	16/47 (34 [21–49])	45/66 (68 [56–79])	27/96 (28 [19]–[38])
Percent difference (95% CI)	47% (28–65)	70% (49–91)	77% (68–86)	46% (26–66)	32% (−1 to 65)	22% (1 to 43)	27% (6–49)	21% (−4 to 46)
Adequate oxygen prescriptions								
Survey 1	0/8 (0)	0/36 (0)	0/17 (0)	0/30 (0)	1/21 (5 [0–24])	0/17 (0)	0/8 (0)	0/52 (0)
Survey 4	3/33 (9 [2]–[24])	38/49 (78 [63–88])	6/25 (24 [9]–[45])	19/51 (37 [24–52])	13/90 (14 [8]–[23])	1/18 (6 [0–27])	1/27 (4 [0–19])	0/60 (0)
Percent difference (95% CI)	9% (−12 to 30)	78% (64–92)	24% (3–45)	37% (19–55)	8% (−23 to 39)	6% (−6 to 17)	4% (−10 to 18)	0%
Pneumonia episodes with a severity classification								
Survey 1	15/97 (15 [9]–[24])	19/189 (10 [6]–[15])	8/100 (8 [4]–[15])	5/137 (4 [1]–[8])	5/18 (28 [10–53])	7/140 (5 [2]–[10])	15/78 (19 [11]–[30])	10/146 (7 [3]–[12])
Survey 4	76/81 (94 [86–98])	204/211 (97 [93–99])	106/111 (95 [90–99])	151/160 (94 [90–97])	14/85 (16 [9]–[26])	77/111 (69 [60–78])	70/145 (48 [40–57])	170/181 (94 [89–97])
Percent difference (95% CI)	78% (69–88)	87% (82–91)	87% (81–94)	90% (86–96)	−12% (−31 to 9)	64% (56–73)	29% (16–42)	87% (82–92)
Malaria episodes with a severity classification								
Survey 1	46/214 (21 [16]–[28])	18/219 (8 [5]–[13])	9/211 (4 [2]–[8])	14/220 (6 [4]–[10])	5/103 (5 [2]–[11])	4/185 (2 [1]–[5])	2/249 (1 [0–3])	3/142 (2 [0–6])
Survey 4	176/184 (96 [92–98])	194/200 (97 [94–99])	163/196 (83 [77–88])	229/243 (94 [91–97])	22/212 (10 [7]–[15])	56/105 (53 [43–63])	60/273 (22 [17]–[27])	100/127 (79 [71–85])
Percent difference (95% CI)	74% (68–81)	89% (84–93)	79% (73–85)	88% (84–92)	6% (−1 to 12)	51% (43–59)	21% (16–26)	77% (69–84)
Dehydration episodes with a severity classification								
Survey 1	27/57 (47 [34–61])	25/42 (60 [43–74])	21/57 (37 [24–51])	27/41 (66 [49–80])	7/10 (70 [35–93])	17/35 (49 [31–66])	33/48 (69 [54–81])	40/73 (55 [43–66])
Survey 4	57/58 (98 [91–100])	115/119 (97 [92–99])	109/110 (99 [95–100])	101/102 (99 [95–100])	28/41 (68 [52–82])	58/62 (94 [84–98])	58/68 (85 [75–93])	130/141 (92 [86–96])
Percent difference (95% CI)	51% (37–64)	37% (27–48)	62% (53–72)	33% (23–43)	−2% (−35 to 32)	45% (30–60)	17% (1–32)	37% (27–48)
**Outcome of care indicator**								
Vitamin A administered on admission								
Survey 1	6/246 (2 [1]–[5])	24/330 (7 [5]–[11])	5/261 (2 [1]–[4])	2/293 (1 [0–2])	6/121 (5 [2]–[10])	21/285 (7 [5]–[11])	74/276 (27 [22]–[32])	0/323 (0)
Survey 4	26/230 (11 [8]–[16])	152/312 (49 [43–54])	232/309 (75 [70–80])	9/307 (3 [1]–[5])	0/271 (0)	44/198 (22 [17]–[29])	0/341 (0)	12/337 (4 [2]–[6])
Percent difference (95% CI)	9% (4–13)	41% (35–48)	73% (68–79)	2% (0–4)	−5% (−8 to −2)	15% (9–21)	−27% (−32 to −22)	4% (2–6)
Provider initiated HIV testing among unknown HIV								
Survey 1	8/240 (3 [1]–[6])	2/330 (1 [0–2])	10/261 (4 [2]–[7])	3/293 (1 [0–3])	0/121 (0)	2/284 (1 [0–3])	3/276 (1 [0–3])	5/322 (2 [1]–[4])
Survey 4	53/227 (23 [18]–[29])	86/312 (28 [23]–[33])	83/307 (27 [22]–[32])	42/301 (14 [10]–[18])	2/269 (1 [0–3])	12/193 (6 [3]–[11])	16/340 (5 [3]–[8])	10/333 (3 [1]–[5])
Percent difference (95% CI)	20% (14–26)	27% (22–32)	23% (17–29)	13% (9–17)	1% (−1 to 2)	6% (2–9)	4% (1–6)	1% (−1 to 4)
Age appropriate documentation of immunisation status								
Survey 1	17/246 (7 [4]–[11])	28/330 (8 [6]–[12])	12/261 (5 [2]–[8])	13/293 (4 [2]–[7])	0/121 (0)	9/285 (3 [1]–[6])	157/276 (57 [51–63])	114/323 (35 [30]–[41])
Survey 4	58/230 (25 [20]–[31])	235/312 (75 [70–80])	220/309 (71 [66–76])	129/307 (42 [36–48])	2/271 (1 [0–3])	47/198 (24 [18]–[30])	230/341 (67 [62–72])	72/337 (21 [17]–[26])
Percent difference (95% CI)	18% (12–25)	67% (61–72)	67% (61–73)	38% (31–44)	1% (−1 to 2)	21% (15–26)	11% (3–18)	−14% (−21 to −7)
Mean proportion of discharge counselling tasks performed (total tasks [range] = 4 [0–4])^a^								
Survey 1	1.52 (1.12–1.93)	0.36 (0.13–0.59)	1.21 (0.86–1.56)	2.00 (1.53–2.47)	1.30 (0.94–1.66)	0.97 (0.43–1.50)	0.92 (0.68–1.16)	1.64 (1.22–2.06)
Survey 4	3.55 (3.22–3.87)	3.02 (2.63–3.41)	3.12 (2.77–3.47)	0.85 (0.45–1.26)	3.36 (3.03–3.69)	0.64 (0.17–1.10)	2.64 (2.20–3.08)	1.45 (0.91–2.00)
Difference (95% CI)	2.03 (1.51–2.55)	2.66 (2.20–3.11)	1.91 (1.42–2.40)	−1.15 (−1.76 to −0.53)	2.06 (1.58–2.54)	−0.33 (−1.02 to 0.36)	1.72 (1.22–2.22)	−0.19 (−0.87 to 0.49)

a Average scores (95% CI).

The proportion of admissions treated in line with clinical guidelines was substantially higher in intervention compared to control sites for prescription of twice rather than thrice daily quinine, once rather than thrice daily gentamicin, appropriate quinine and gentamicin dose/kg body weight, and the proportion of severely dehydrated children with correct intravenous fluid volumes (Table 3). There were no differences in proportions receiving possibly toxic gentamicin doses although this practice was relatively uncommon.

**Table 3:** Average performance in control and intervention hospitals at baseline and 18 mo follow-up and adjusted difference (95% CI) at 18 mo.

Indicator of Quality of Care	Intervention		Control		Adjusted Difference between Groups at 18 mo (%)^a^	95% CI		*p*-Value
	Survey 1	Survey 4	Survey 1	Survey 4				
*Process indicators*								
Child's weight documented	59.3	84.5	21	63.2	22.8	−4.05	49.7	0.080
Child's temperature documented	11.9	71.9	25.1	46.6	26.5	−4.49	57.5	0.080
Average assessment score	0.24	0.94	0.32	0.65	0.29	0.05	0.54	0.030
Proportion of pneumonia episodes with a severity classification	9.29	95.1	14.7	57.0	38.57	9.87	67.3	0.017
Proportion of gentamicin prescriptions with once daily dose	1.85	89.2	3.54	74.4	17.05	8.04	26.1	0.004
Proportion of gentamicin prescriptions with daily dose <4 mg/kg	24.9	2.16	23.4	8.99	−6.77	−11.9	−1.59	0.019
Proportion of gentamicin prescriptions with daily dose ≥10 mg/kg	3.78	6.25	7.15	9.82	−3.54	−11.1	4	0.294
Proportion with adequate oxygen prescriptions	0	37.0	0	2.31	35.1	7.32	62.8	0.021
Proportion of malaria episodes with a severity classification	10.1	92.5	2.48	41.1	52.1	26.2	78.0	0.003
Proportion of severe malaria with quinine loading	4.20	91.9	14.8	66.7	26.3	−3.66	56.3	0.075
Proportion of severe malaria with twice daily quinine maintenance dose	0.39	87.8	9.95	45.7	42.6	25.1	60.2	0.001
Proportion of severe malaria with quinine daily dose ≥40 mg/kg	7.33	1.02	14.1	7.46	−6.53	−12.9	−0.2	0.045
Proportion of dehydration episodes with a severity classification	52.4	98.3	60.5	84.8	14.4	4.27	24.6	0.013
Correct intravenous fluid prescription	7.32	67.2	15.0	40.6	29.9	10.9	48.9	0.008
*Outcome indicators*								
Proportion with vitamin A administered on Admission	3.08	34.5	9.78	6.45	28.3	−7.11	63.6	0.098
Age appropriate documentation of immunization status	6.11	53.4	23.8	28.3	25.8	7.29	44.4	0.014
Provider Initiated HIV testing among unknown HIV	2.2	23.0	0.84	3.67	19.4	12.3	26.4	0.001
Mean number of discharge counselling tasks performed (total tasks = 4)	1.27	2.64	1.21	2.02	0.61	−1.48	2.71	0.50

A negative difference indicates a reduction in the proportion of case records showing inappropriate practice.

a Adjusted difference between intervention arms obtained from linear or logistic regression analysis of hospital summary data adjusting for child's sex, illness outcome, and hospital factors (size, malaria endemicity, HIV prevalence).

### Secondary Effectiveness Measures

#### Outcome indicators

At baseline key child health policy interventions were rarely implemented. Vitamin A was prescribed only in H7 to 27% of admissions (Table 2). Health workers rarely documented missed opportunities for immunization (<9% across six sites) or offered PITC for HIV at baseline (all sites fewer than 4%).

At 18 mo the proportion of children offered PITC for HIV was significantly higher in intervention sites (adjusted difference, 19.4%; 95% CI 12.3%–26.4%), as was checking vaccination status (25.8%; 7.29%–44.4%]). Although, prescription of Vitamin A and counselling improved in some hospitals, differences between groups did not attain statistical significance (Table 3).

#### Structure indicators

**Figure 3:**
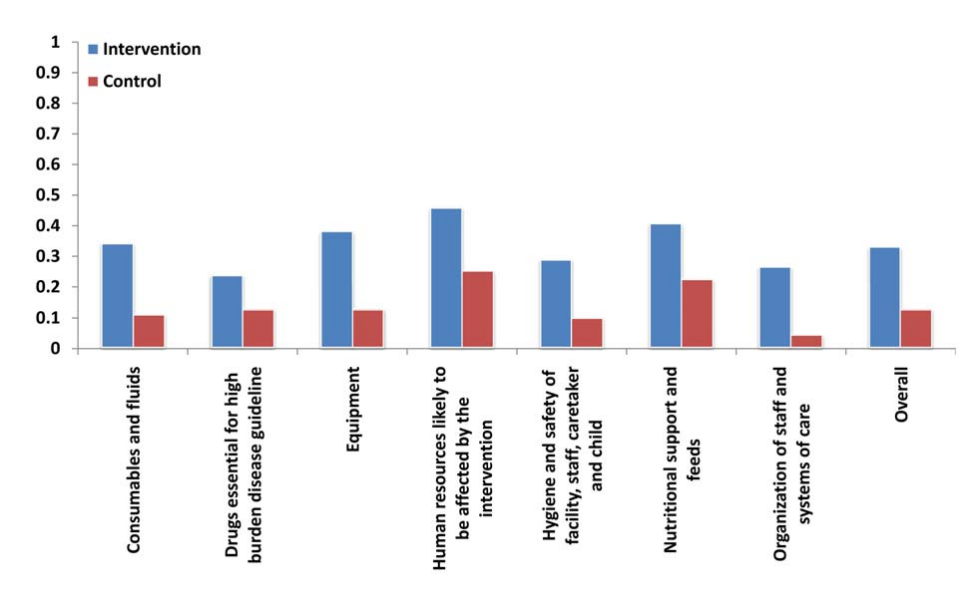
Average change from baseline to 18 mo postintervention in proportion of structure items available, for each major domain and combined, for hospitals in the intervention and control groups.

Changes between baseline and 18 mo were positive in both groups for all domains. Improvements in intervention hospitals were, however, consistently greater than in control hospitals (Figure 3), with the mean difference of difference analysis showing a 21% greater overall improvement (*p* = 0.02, based on a simple *t*-test).

#### Performance within intervention sites during surveys 5 and 6

**Figure 4:**
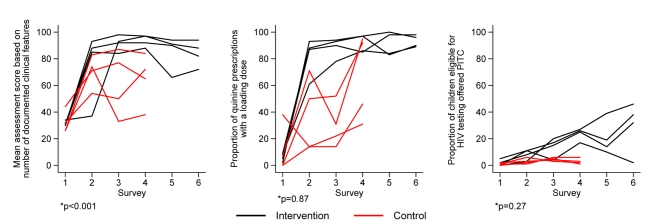
Intervention effect on processes of care. (a) Documentation of essential clinical signs for malaria, pneumonia, or dehydration; (b) proportion of children receiving loading dose quinine, and outcome of care; (c) the proportion of children eligible for HIV testing offered PITC during survey 1 through survey 6 (baseline to 30 mo follow-up).

For most process indicators with improvement, and based on tests for trend between survey 4 and survey 5 or survey 6, no major decline in performance was noted even 12 mo after withdrawal of intervention and in the face of continuing staff turnover (Figures 4 and S1).

## Discussion

We tested an approach to implementing clinical guidelines for management of illnesses that cause most deaths in children admitted to district hospitals in Kenya. Despite their modest success in developed countries [15], we used a multifaceted approach reasoning that deficiencies in knowledge, skills, motivation, resources, and organization of care would all need to be addressed. The intervention design was guided by experience in the setting [7],[8] and theories of change and culture of practice [13],[15],[27]–[29]. Our baseline data and other reports [7]–[10] suggest that the simple availability of authoritative WHO and national guidelines—for periods of more than 15 y—are currently having little impact on hospital care for children. So what did our interventions achieve?

The full intervention package resulted in significantly greater improvements in almost all primary and secondary effectiveness measures. Within specific hospitals performance of certain indicators, e.g., recording child's weight in H3, were already high at baseline. For these specific hospitals there was limited scope for improvement, but there remained significant potential for improvement at the group level since performance for most indicators was below the projected level of 50% at baseline. Substantial, clinically important changes occurred in processes of care despite very high staff turnover amongst the often junior clinicians responsible for much care in each site. Indeed, of 109 clinical staff involved in admitting patients sampled at survey 4 from intervention hospitals only nine (8.3%) had received any specific formal or even ad hoc training. At survey 6 this proportion had reduced to 4.4% (four out of 91) reflecting the typically high turnover of junior clinicians in such settings. As the training and guidelines were not being provided in preservice training institutions and as formal orientation periods are absent [14], we infer, but cannot confirm, that new staff learned correct practices more commonly from established clinicians or the facilitator in intervention hospitals. Improvement in structure indicators occurred without any direct financial inputs reflecting probably a small generalized improvement in resource availability and use of funding from user fees (total hospital incomes varied from US$57 to US$100 per bed per month [19]) that we feel was in part, in response to hospital feedback and the advocacy of the facilitator [14].

Improvements in quality of care thus occurred across a set of common, serious childhood conditions and over a prolonged period. These data are a major addition to reports from sub-Saharan Africa indicating that financial incentives can improve malaria-specific care and fatality [30] and that implementation of WHO guidelines can improve emergency triage assessment and treatment of children [31]–[33] and hospital care and outcomes for severe malnutrition [34]. They also complement evidence from middle-income settings where a multifaceted intervention resulted in substantial improvements in two key obstetric practices [35]. Our data however, to our knowledge, represent the first major report examining national adaptation and implementation of a broad set of rural hospital care recommendations. They are relevant to many of the 100 countries with IMCI programmes where rural hospitals have important roles supporting primary health care systems [36] and in helping to reduce child mortality [37],[38].

However, while change in simple process indicators was reasonably consistent in intervention sites, in control (partial intervention) sites, changes were more varied, even within hospitals (notably site H8). Certain indicators, e.g., PITC for HIV, also improved only in three of four intervention sites and steadily but slowly. Thus, while the full intervention may promote consistency, there was still substantial evidence of variation across indicators, across sites, and across time. Such variability is consistent with emerging debates drawing on theories of complexity, chaos, and change emphasizing the effect of interactions with contexts [39]–[41] and suggesting that understanding can be informed by parallel qualitative enquiry [42]. Data collected during this study on barriers to use of guidelines [18] and views on supervision, feedback, and facilitation [14] together with published literature [43] suggest to us that poor or slow uptake may be associated with a requirement for greater personal or organizational effort to change, the view that a task is not directly related to care of the immediate illness, or, in intervention sites, an area unlikely to be subject to local evaluation.

### Limitations

Our study has limitations. Hospitals were not selected at random from a set of all eligible hospitals for logistic reasons and, because random selection of a small number of clusters may not have produced balance nor guaranteed representativeness at baseline. Hospitals assented to participation and randomization, but we were not able to engage communities in this process [44], and they and survey teams were aware of intervention allocation. The latter is a potential problem with results based largely on retrospective review of records. The discrepancy between documentation and performance presents a particular threat at baseline before efforts in all sites to improve clinical notes. Prescription data are less susceptible to this limitation however, and improved prescribing paralleled improvement in assessment indicators. Efforts to minimize possible observation bias at the point of data collection included the use of structured inventory forms, standard operating procedures, and extensive training in survey methods. With only four hospitals per group, attempts to adjust for baseline imbalance may also have only limited success. However, to facilitate scrutiny we report on the context of intervention [19],[20], its delivery and adequacy [12], the views of intervention recipients [18], and detailed site-specific data (see Tables S1, S2, S3, S4, S5) and suggest that all are considered for a complete interpretation of this study of a complex intervention.

### Replication and Scaling Up

Demonstrations that a similar intervention package is effective in other settings would strengthen the evidence supporting widespread adoption. While there are few studies of this nature reported, we note the recently reported success of multifaceted interventions in middle- and high-income countries [35],[45]. However, standardizing complex interventions may be difficult, if not impossible, given the important role of context in shaping mechanisms and outcomes [46]. For this reason, future reports will attempt to provide detailed insight into how and why this intervention met with general but varying degrees of success. If our results are deemed credible, however, the data we present have a number of implications. Firstly, current efforts to implement and scale up improved referral care in low-income settings need to go beyond the existing tradition of producing and disseminating printed materials even when linked to training [15]. Instead broader health system efforts, guided by current understanding of local contexts and capabilities and theories of change, are required.

Within Kenya it would obviously be a mistake to consider that the intervention package tested can be scaled up simply by aiming for much broader coverage with the training course we designed. Effectiveness has been demonstrated only for the multifaceted intervention. Thus, scaling up should aim to provide all inputs not just guidelines, job aides, and introductory training. However, providing regular support supervision and performance feedback related to child and newborn care at first referral level are not routine. Resources and systems for supervision need strengthening and supervisors themselves will need training and organizing. Routine information systems are inadequate to generate the data required to evaluate care, and capacity for conducting and disseminating analyses as part of routine feedback is largely absent. The role of facilitators is also not one that currently exists. Although the roles required could perhaps be played by senior departmental staff, the lack of human resources means such tasks cannot simply be added to already busy jobs [19]. Furthermore the skills or desire to facilitate change are not necessarily present amongst such mid-level managers.

Countries other than Kenya considering adopting the approach may have similar limitations. In addition they may need to tailor some intervention components to their particular setting. For example, the detail of a clinical guideline or job aide or approach to training may need to reflect available resources or local evidence. However, such adaptation would need to be complemented by careful consideration of how systems can be made ready to support implementation of new practices and improved quality of care. We would suggest this includes due attention to influencing the institutional culture and context of rural hospitals although willingness to invest in more integrated approaches often seems lacking [47]. Finally, before making decisions on implementation, policy makers increasingly require carefully collected and reported cost-effectiveness data. Such a report is in preparation. Considering only the financial costs of specific inputs, for example the typical 5-d training course for 32 participants at approximately US$5,000 [13] or the annual cost of a facilitator at less than US$5,000 [18], while of some value, are insufficient for prioritizing resource use.

### Conclusion

Our findings provide strong evidence that a multifaceted intervention can improve use of guidelines and, more generally, the quality of paediatric care. Cost data will help determine whether this implementation model warrants wider consideration as one approach to strengthening health systems in low-income settings.

## Supporting Information

Figure S1 Effect of intervention on the processes and outcome of care within each hospital during survey 1 through survey 6 (baseline to 30 mo follow-up).
(TIF)

Table S1 Demographic characteristics of all 8,205 children aged 2–59 mo by hospital and survey.
(XLS)

Table S2 The main diagnoses among all 8,205 study participants aged 2–59 mo by hospital during each survey.
(XLS)

Table S3 Difference-in-difference analysis of intervention effect on process and outcome measures of quality of care.
(XLS)

Table S4 Changes for process indicators by hospital during each survey.
(XLS)

Table S5 Changes for outcome indicators by hospital during each survey.
(XLS)

Text S1 CONSORT checklist.
(PDF)

Text S2 Trial protocol.
(PDF)

## Abbreviations

CI: confidence interval
IMCI: Integrated Management of Childhood Illnesses
PITC: provider initiated testing and counselling
