# Supplementary figures and images for: A Multifaceted Intervention to Implement Guidelines and Improve Admission Paediatric Care in Kenyan District Hospitals: A Cluster Randomised Trial
Source: PLoS Med. 2011 Apr 5;8(4):e1001018. doi: 10.1371/journal.pmed.1001018 (PMC3071366; doi:10.1371/journal.pmed.1001018)

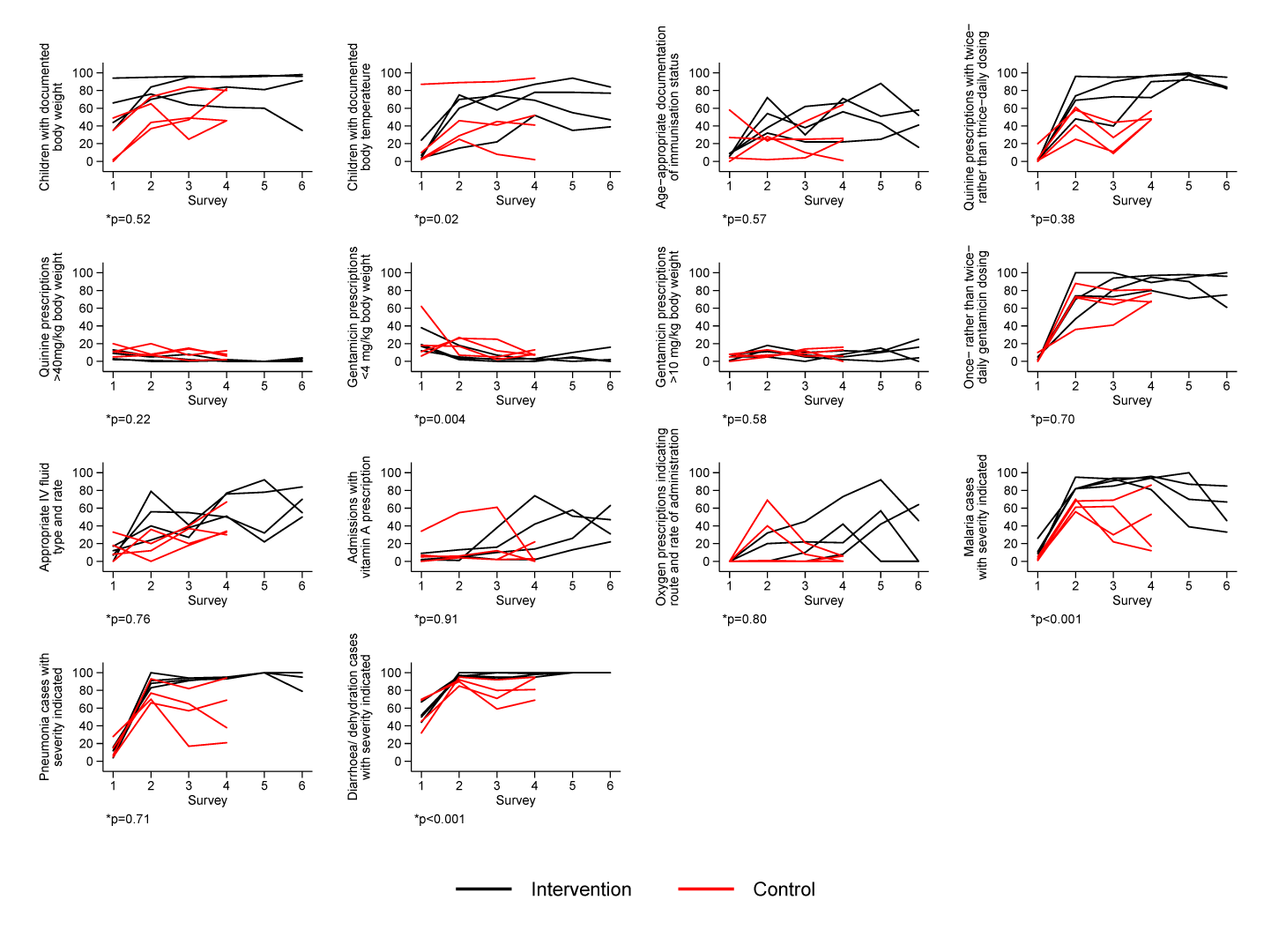

Supplement: Figure S1 — Effect of intervention on the processes and outcome of care within each hospital during survey 1 through survey 6 (baseline to 30 mo follow-up). (TIF) [file pmed.1001018.s001.tif]
