# Supplementary material for: A Multifaceted Intervention to Implement Guidelines and Improve Admission Paediatric Care in Kenyan District Hospitals: A Cluster Randomised Trial
Source: PLoS Med. 2011 Apr 5;8(4):e1001018. doi: 10.1371/journal.pmed.1001018 (PMC3071366; doi:10.1371/journal.pmed.1001018)
Supplement: Text S2 — Trial protocol. (PDF) [file pmed.1001018.s008.pdf]

1) Assessing the impact of an intervention to implement the referral care component of Integrated Management of Childhood Illnesses (IMCI) in district hospitals.

2) INVESTIGATORS.

Principal Investigator: Dr. M English, 1*

**Co- investigators:**	**Kenya**	**International Collaborators:**
	Dr. A. Wamae, 2, ¶	Prof. L. Gilson, A¶
	Dr. P. Migiro, 2 ¶	Dr. A. Rowe, C ¶
	Prof. A. Wasunna, 3 ¶	Dr. M. Weber, D ¶
	Dr. F. Were, 3 ¶	Dr. K. Hanson, B, ¶
	Prof. F. Esamai, 4 ¶	
	Dr. A. Muriithi, 5 ¶	
	Prof. R.W. Snow, 1*	
	Dr. G. Fegan, 1	
	Dr. N. Peshu, 1	
	Mr. Patrick Mbindyo. 1*	

1) Centre for Geographic Medicine Research – Coast, KEMRI / Wellcome Trust Collaboration, P.O. Box 230, Kilifi, Kenya. (* and P.O. Box 43640, Nairobi)
2) Division of Child Health, Department of Preventive and Promotive Health, Ministry of Health, Old Mbagathi Road, Nairobi.
3) Academic Department of Paediatrics and Child Health, College of Health Sciences, University of Nairobi, Nairobi.
4) Department of Paediatrics, Faculty of Health Sciences, Moi University, Eldoret.
5) World Health Organisation, Kenya Country Office, Nairobi, Kenya.

A) Centre for Health Policy, University of Witswatersrand, Johannesburg, South Africa.
B) Health Economics and Financing Programme, London School of Hygiene and Tropical Medicine, London, UK.
C) Malaria Branch, Centres for Disease Control, Atlanta, USA.
D) Department of Child and Adolescent Health, World Health Organisation, Geneva, Switzerland.

¶ CVs supplied in Appendix 4.

3) ABSTRACT:
We intend to conduct a unique public health efficacy study of an intervention to improve care for children in district hospitals in Kenya. The intervention has been developed, based on the referral care component of the IMCI strategy, with the Ministry of Health. It comprises, training, guidelines, job aides, supervision and quality improvement activities and will be delivered over 1.5 years to four intervention hospitals. Four control hospitals will receive guidelines and information from regular surveys. Random selection will be used to decide allocation to the hospital groupings. The impact of the interventions in hospitals from both groups will be monitored over 2.5-3.0 years (extending before and after intervention) with performance assessed against the guidelines provided and pre-defined standard criteria. Assessments will include:

1) Process measures of the quality of care, representing proximate impacts of the intervention,
2) Paediatric inpatient mortality and the adequacy of resources and the environment (outcomes with a complex causal pathway)
3) Exploration of factors at hospital and health worker levels (including institutional and person-specific factors such as motivation) that may affect the delivery of care
4) The costs and cost-effectiveness of the intervention

Qualitative studies will be used to describe: each hospital’s context and the broader institutional response to intervention.

Results will critically inform the debates on scaling-up and improving the delivery of evidence based hospital care for children in Kenya and elsewhere.

4) INTRODUCTION:

Under 5 mortality in most of sub-Saharan Africa including Kenya remains >100/1000 live-births and has remained unchanged for a decade or has risen in some countries including Kenya [1]. Better delivery of ‘close-to-client’ health services is required if major improvements in child survival are to be realised and such services are likely to be at least as cost effective as many new vaccines [2]. Appropriately therefore the delivery of health services at the community level and through primary care units (PCUs) has been the subject of considerable global research and now calls to action [3-6]. However, district hospitals that provide referral care, and the complex environments in which they operate, have been largely ignored by researchers [7-9]. For the reasons outlined it is time that research contributed to better performance of hospitals in low-income countries.

1) ***Referral care is commonly required.*** In sub-Saharan African countries between 6% and 20% of children assessed at primary care facilities may require referral [10]. In Kenya, using the conservative value of 6%, it can be crudely estimated that 0.6 million children aged less than 5 years, equivalent to 1 in 8 of the under 5 population, might require referral care annually (ME, unpublished data).
2) ***Effective hospital care can improve child survival.*** It has been estimated that the presence of hospital care confers a considerable child survival advantage for the population served by the hospital [11].
3) ***District hospital care can be highly cost effective.*** Data on the costs or cost-effectiveness of hospital services in low-income countries are extremely rare. Kenyan data suggest that the cost per child life saved by hospital care may be as low as \$105 [7] indicating that basic hospital care may be a relatively ‘good buy’.
4) ***Hospitals are an established part of many health systems.*** The majority of countries, even in Africa, have a sizable hospital sector consuming considerable resources. However, it has been demonstrated that relatively poor quality services are frequently offered [12-14], limiting their effectiveness and resulting in a poor return for this investment.

If services can be relatively cost-effective and are frequently needed questions on how to develop equitable health systems [15] should, therefore, include ‘how can basic hospital services best be provided to support primary care, particularly for the poor?’ This question is particularly relevant to aims to reduce child mortality by two-thirds by 2015, one of the

Millenium Development Goals (MDGs) [16].

In Kenya there are just over 100 government hospitals providing basic referral care, 70 of which are district hospitals (DHs) that serve and supervise networks of government PCUs (n = 1944 nationally) [17]. In common with many countries in sub-Saharan Africa these hospitals face problems with infrastructure, equipment, personnel, supplies of resources [12,14,18] and, sometimes, poor management [19]. However, first line therapeutics for the most common diseases are widely available [14] and clinical staff (predominantly clinical officers) and nursing staff are present to assess and administer treatment to children 24 hours a day. The basic requirements for effective admission care for malaria, diarrhoea and other serious infectious diseases, the major childhood killers, are thus present, although what is offered is often inadequate or inappropriate [13]. As more than two-thirds of deaths from these key diseases in children occur within 48 hours of hospital admission [20,21] there is a clear rationale for improving immediate care, even in the absence of broader health system improvements.

Kenya, together with over 100 other low or middle income countries, is currently involved in efforts to scale-up the primary care component of the WHO/UNICEF Integrated Management of Childhood Illness (IMCI) programme. This approach combines previous, distinct Acute Respiratory Infection, Diarrhoeal Disease and other 'vertical' programmes into a country-adapted integrated case management package. A central theme of the training for primary care health workers is identification of severely ill children who need referral to hospital. In theory the IMCI strategy includes evidence-based recommendations for appropriate clinical management of these very sick children at the hospital or referral-care level. However, the means to translate these recommendations into actual changes in improved health care delivery have not been examined. As Kenya is implementing the primary care component of IMCI countrywide it is an opportune time to investigate how best to implement the hospital care component [22].

Choosing intervention strategies.

The functioning of district hospitals, as part of complex health systems, is affected by a wide variety of factors (see appendix 1, figure 1). These include effective health policy and regulation and the provision of adequate human, capital and consumable resources [23]. At a local level, resource allocation, individual health worker motivation, organisational structures, institutional and personal values and trust will impact on hospital performance [24-26]. The

demand for services, reflected by the effectiveness of referral, is also likely to be a key determinant of efficiency, equitable distribution of resources and population health benefits. Given this complexity multiple interventions targeting health system constraints above, within and below the district hospital level are likely to be necessary to maximise health gains from better performing hospitals.

Health workers, however, are central to a health system's functioning [27] and interventions delivered primarily at this sector in a district hospital setting may both improve patient outcomes and stimulate additional, local health system improvements (see below). The ability to influence health worker practices and the interplay between health workers and their immediate context in a low-income country, facilitated by an increase in the availability of information, is a central theme of the research proposed in this application as even with currently available resources it is likely that health care delivery could be considerably improved.

How to implement best practice.

While it is obviously an oversimplification it is nonetheless useful to consider implementation of best practice care in two parts. First, ensuring that the environment and resources are conducive (see appendix 1, figure 1) and second, changing health workers' behaviour. For the latter, evidence has accumulated from developed countries that the provision of written, expert guidelines and training alone often have limited effects on changing practices, with more marked effects apparent if these approaches are combined with job aides, feedback and supervision [28-31]. However, such strategies focus predominantly on what should be done. Quality improvement (or assurance) approaches include the slightly broader question of how barriers to the introduction of care can be overcome at a local level to facilitate best practice [32]. Therefore, quality improvement involves health workers at all relevant levels in a participatory approach, empowering the 'grassroots' of the health sector to solve local problems [33].

Assessing health worker and hospital performance.

The rationale for an intervention to promote better case management is that such care is on the causal pathway to better outcomes. Process indicators reflecting the degree to which best practice care is provided are therefore valid and appropriate endpoints. Such process measures have many additional, desirable properties: they may permit between health worker and cross-

centre comparisons; they can target the most desired attributes of service delivery; they are relatively cheap; they can rapidly incorporate new elements; and they provide results that are intrinsically meaningful to service providers [34,35]. At the individual or hospital level interpretation of process and outcome measures is affected by the degree to which inputs (resources) are available that therefore need to be assessed too. Interpreting aggregate performance or mortality data is complicated by potential variations in case-mix and case-severity. These difficulties are compounded in cross-centre comparisons by differences in the hospitals themselves and the populations they serve, even when substantial efforts are made to collect large, high quality datasets that permit adjusted analyses [36-38]. The problems with aggregate hospital performance indicators [34] suggest they must be interpreted cautiously but does not, however, preclude meaningful assessment of performance in well-defined, specific domains [39].

We have presented evidence that improvement in health care delivery for sick children in hospital is: necessary, possible even with existing resources, may theoretically be achieved by interventions delivered through local health workers, and may be plausibly monitored. We now present evidence underlying the choice of proposed intervention and monitoring strategies. It is proposed that two groups of hospitals are studied. One group will receive a comprehensive package of interventions and another will receive a more traditional intervention of written guidelines and written feedback on performance assessment. The latter group will provide information on any changes in Kenya's hospital sector during the period of study that might be due to new government policies or initiatives or major economic changes.

The four-part model intervention provided to intervention hospitals has been designed to reflect the emerging consensus that multiple approaches are required to make major changes in health care practice. As defined the research team represents an intrinsic part of the intervention taking the role of a regional or national paediatric quality assurance programme. A role that must be assumed in the absence of any existing supervisory structures in Kenya and that is justified if we wish to address the question of whether and to what degree governments like Kenya should invest in such activities.

The research proposed therefore explores the potential of methods regarded as central to health systems improvement in developed nations to improve paediatric care in district hospitals in Kenya. Comprehensive descriptions of the context within which care is delivered

will provide a framework to help interpretation of the results. The work proposed will provide much needed information on how the referral care component of the IMCI programme might be scaled-up. Proof in principle that the proposed intervention works would argue strongly both for its inclusion as an integral part of health system strengthening and for its consideration as a stand-alone approach pending broader health sector reforms in developing countries.

5) JUSTIFICATION.

The Government of Kenya aims to provide "sustainable, quality health care that is acceptable, affordable and accessible to all Kenyans" [40]. One of the aims of the introduction of IMCI to Kenya is to facilitate this through the provision of appropriate care for children and in so doing reduce childhood mortality in the country. Inpatient care at the first referral level is broadly included in the IMCI approach but so far less attention has been given to this aspect than to that of outpatient care although indicators in Kenya and elsewhere suggest there are considerable problems with referral care. We intend to assess the impact of an intervention package aimed at improving the quality of care for severely ill children in district hospitals. If the intervention proves effective and can be provided at reasonable cost there would be a strong argument for supporting the scaling up of such a programme in support of current, national efforts to implement the primary care component of IMCI. Lessons learned in the implementation and evaluation of the intervention may also be invaluable to other countries attempting to lower their childhood mortality rates.

6) NULL HYPOTHESIS:

The statement of a statistically meaningful null hypothesis is not appropriate for this large, primarily observational study.

7) OBJECTIVES.

General Objectives.

To investigate the degree to which the quality of paediatric inpatient care at district hospitals can be improved by an intervention comprising evidence based guidelines, training, supervision and facilitation of local problem solving and at what financial cost.

To explore those factors that hinder or promote the delivery of quality care for children admitted to hospital.

Specific Objectives.

1) To evaluate the degree to which an intervention comprising explicit standards and evidence based guidelines for paediatric care, training, supervision and feedback will improve the quality of care for hospitalised children. The quality of care will primarily be assessed against pre-defined process standards.
2) To examine factors at the hospital and health worker levels that affect the successful delivery of hospital care for children.
3) To measure the costs of the intervention and the cost per additional child receiving best practice admission care.
4) To use the 10-item motivation tool to explore, amongst Clinical Officers, the influence of employer type and / or years of service on motivation score

8) DESIGN AND METHODOLOGY.

a) Study Site.

Kenyan hospitals to be studied will be chosen together with the Division of Child Health at the Ministry of Health (MoH). It will not be possible to take a random sample of all MoH district hospitals. Instead a short-list of hospitals with a paediatric workload of at least 1200 admissions per annum, a maternity workload of at least 1000 deliveries per annum and in which less than 10% of paediatric admissions are referred from another hospital will be constructed. The factors that influence hospital performance in the delivery of paediatric care are unknown – part of the purpose of this research is to explore these. Therefore study sites

cannot be selected on the basis of known confounders at the hospital or population level. However, it is likely that both hospital and population factors influence performance and the selection of study sites will attempt to address those that are considered most plausible and / or important. Thus, districts and their respective hospitals on the short-list will be classified on the basis of available data as:

1) Situated in, or serving a population exposed to, endemic malaria with the potential for year round transmission of disease (Yes or No).
2) Serving a population with antenatal prevalence of HIV ≥ 10% (Yes or No).
3) Representative of the lowest tertile in the national distribution for child health and development indicators at the district level including; EPI vaccine coverage, proportion of children with stunting and primary school enrolment of girl children (Yes or No).
4) Serving an urban / peri-urban population of >50,000 in the immediate vicinity of the hospital (Yes or No).
5) Able to routinely allocate a medical officer or paediatrician to daily, weekday supervision of paediatric inpatient care (Yes or No).

Eight hospitals / districts from the short-list will be chosen to ensure at least two hospitals meet each positive and negative criteria above to reflect the diversity of hospital settings in Kenya (a single hospital may fulfil multiple criteria) and improve the generalisability of subsequent results.

Having identified eight potential sites all possible combinations of allocation to two groups of four (intervention and control) will be listed. The theoretical allocations to two groups of four sites that preserve balance between the groups with respect to the classifying factors listed above (1 to 5) will be retained. From this list of group combinations that represent ‘balanced allocations’ the final group allocation will be chosen at random – representing a process of ‘restricted randomisation’. This process will help ensure that the intervention and control hospitals are similar with respect to factors that might influence performance and will prevent the investigators from introducing selection bias through purposeful selection.

To enable a comparative analysis of influences on health worker motivation, we shall select health workers for interview from a sample of faith-based district-type hospitals that have a high number of Clinical Officers. Candidate hospitals include AIC Kijabe, PCEA Chogoria, PCEA Kikuyu, St. Elizabeth, Mumias, among others.

b) Study Population.

The primary unit of analysis will be the hospital. Within each site data will however be collected by / from:

1) Observation on the availability of key resources (eg. oxygen delivery devices) and hospital characteristics (eg. the practice of outpatient triage).
2) Senior hospital staff and administrators on the level of resource provision (eg. drug availability) and on the local decisions about resource allocation where these are hospital controlled.
3) Hospital case records of children admitted.
4) Interviews and focus group discussions with health workers
5) Interviews with the caretakers of admitted children.

c) Sampling.

i) Sample size:

<u>Total number of hospitals.</u>

The primary design corresponds to a series of detailed case studies of how hospitals respond to a broad based approach to improve paediatric care (referred to as the intervention group of hospitals) or to the provision of guidelines and written feedback on the local quality of care only (referred to as the control group). The sample size of four hospitals per group was selected bearing in mind the following considerations:

a) To permit as wide a range of hospitals as possible to be included to help in assessing the generalisability of findings and to improve the ability to explore the range of factors that might influence changes in performance .
b) To reflect the fact that detailed observations made over a longer period of time – representing a period of opportunity for change - are likely to be more valuable than assessments representing only short time periods.
c) That a law of diminishing returns is likely to operate with regard to the major findings – that successive increases in the number of hospitals are each likely to yield fewer new and major insights while the costs and logistic difficulties associated with the study would continue to rise substantially and threaten the ability to collect high quality data.

For some hospital level indicators (such as the successful operation of outpatient triage)

therefore only 4 observations per group will be available. This sample size is too small for meaningful statistical analysis. However, such changes in practice will be reported together with additional indicators of change to provide a detailed description of events after intervention.

Sample sizes for within hospital findings.

At each site observations will be made spanning a period of 3 years. For intervention sites this period can be divided into six, 6-months periods, for control sites three 12-months periods. Twelve monthly surveys are to be used in control hospitals primarily because frequent surveys might themselves influence a hospital's performance (enhance a Hawthorne effect) and therefore reduce the apparent value of the intervention. At each survey process measures of performance will be made, allowing changes between periods, within a site, to be monitored. Key process measures will reflect overall indicators (e.g. evidence that key assessment tasks are undertaken), common disease specific indicators (e.g. the use of an appropriate type and dose of anti-malarial drug) and uncommon clinical condition indicators (e.g. the appropriateness of blood transfusion). The confidence intervals surrounding a point estimate of 50% for these process indicators (the value providing the most conservative confidence limits) are indicated in the table.

Monitoring within sites will therefore allow changes of more than 10%, 24% and 38% (representing the differences needed between baseline and subsequent estimates to ensure non-overlapping confidence intervals, see table) for overall, common disease and uncommon condition indicators respectively to be plausibly associated with the intervention. If these changes are maintained or increased over successive observation periods and are more pronounced in the intervention sites then the plausibility that the intervention is causally associated with improved performance will be strengthened.

	N for each observation period	Confidence Interval around 50%
Overall process measure.	400	+/- 5%
Common disease indicator – a common disease is estimated to result in 20% of admissions	80	+/- 12%
Uncommon clinical condition indicator – a minimum number of cases will be examined.	30	+/- 19%

ii) Sampling procedure

Hospital selection.

Candidate hospital identification has been described above. Once a hospital has been identified as a potential site, and after group allocation, visits to the Provincial Medical Officer (PMO) and hospital will be arranged. At these visits the PMO and the district hospital management board (DHMB – the board contains lay members from the local community) will be briefed on the nature of the study and the DHMB invited to participate with activities relevant to their group allocation. If the DHMB provide institutional assent then follow-up meetings will be called to explain the study to hospital staff. A key point in the meetings with hospital staff will be to emphasise that all data will remain confidential and that the study data will not be used in any punitive sense.

d) Procedures.

Intervention provided to 'intervention' hospitals.

Clinical Guidelines - Evidence based guidelines for paediatric care, with an essential resource list, and the quality standards being aimed for will be presented to Group 1 hospitals with the results of their respective baseline surveys. Draft forms of these guidelines were adapted to Kenya's context from generic WHO materials [22] at a meeting co-hosted by the PI and the MoH Division of Child Health that included broad representation of Kenya's paediatricians. These have subsequently been used as the basis for simple admission protocols and draft quality standards for district hospital paediatric care as part of this ongoing collaboration. These guidelines, protocols and standards will serve as the 'gold standards' against which actual practices will be compared and used to identify problems with care.

Training and job aides – Health workers in intervention hospitals will be provided with 7 days theory and skills training, including bedside practice, all delivered at the hospital itself by the research team. This training is based on a 3 day training approach developed by the PI around the Kenyan guidelines and job aides, a 1 day newborn life-support training appropriate to Kenya and a 3 day Emergency Treatment and Triage (ETAT) training developed by WHO [41] recently piloted in Kenya. Running two courses in parallel, one in the mornings and one in the afternoons, we have been able to train over 30 health workers from a single hospital (representing more than 50% of all clinical and nursing staff having any responsibility for children) in one training episode. Follow-up training will specifically target, but not be

limited to, clinical staff providing acute admission care (usually numbering less than 15 per hospital) not originally trained. Job aides will include pocket books of protocols, drug doses and fluid guidelines, wall charts and a standardised paediatric admission record.

External Supervisory Feedback - This will be provided in two forms by the research team. The first will take the form of written feedback for hospitals after major surveys at 6 monthly intervals (figure 2). Past experience indicates that this feedback can be provided within 8 weeks of the survey [42]. This written feedback will be reinforced by personal visits from the research paediatrician every 3 months, coinciding with the short, 3 monthly and major 6 monthly surveys. These visits will include bedside discussions and observations of clinical assessment and are intended to mimic hospital supervision from a regional or national level body.

On-site quality improvement. - Our intention is to test whether investing in training and quality improvement yields results in the same way that one would question whether investment in a vaccine yields results. A non-physician health worker at each hospital will therefore be provided by the project for 1.5 years. This input represents a part of the intervention, acknowledging that future health system changes are likely to require strategic investments, including in personnel [27], not simply a re-shuffling of inadequate resources. In addition, local hospital staff will be encouraged to take part in on-site research activities (data collection, data entry, simple analyses etc) specifically by including payment for 'locum work', equivalent to 1 year's full time salary per hospital, in the project budget. This takes advantage of the common practice in Kenya of health personnel working a large proportion of their 6 weeks annual leave, often in private health facilities. Full time, on-site project personnel will receive the same training as the hospital staff and additionally be instructed in basic data entry, how to conduct problem based mortality audit and how to use simple quality improvement tools, skills they will impart to local staff. In collaboration with hospital staff they will undertake systematic monitoring of care for key diseases, assisted by automated performance reports generated by the data entry programme. Results of this monitoring and the mortality audit will be fed back through a local quality of care group to the hospital and will act as an entry point for initiating problem solving activities. Continued monitoring will permit the success of locally developed solutions to be evaluated as part of the quality improvement cycle [32]. Hospitals will be encouraged to tackle critical resource gaps identified through this mechanism through appropriate use of hospital income. At the end of the first

year a limited budget (maximum $5000 per site, from a fund to be established with WHO) will be made available to each intervention hospital to fill remaining non-personnel, capital resource gaps. Documentation of this local information generation, processing and sharing, and any action resulting will be kept as part of a quality improvement diary by on-site personnel.

Input provided to control hospital by study personnel.

Clinical Guidelines – Written evidence based guidelines for paediatric care, approved by the Ministry of Health, with an essential resource list, and the quality standards being aimed for will be presented to Group 2 hospitals with the results of their baseline surveys.

External Supervisory Feedback - This will be provided in written form only after major surveys at 12 monthly intervals (figure 2) within 8 weeks of the survey.

The study will not prevent control (or intervention) hospitals from receiving new assistance, staff, or other interventions delivered by governmental or non-governmental organizations during the study period. The aim is to collect information on changes that may be occurring in the health sector independent of the study-related interventions, these include the implementation of new policies or programmes.

i) Data collection.

After discussion with hospitals and approval of the study data collection will proceed using a range of instruments. The indicators that these instruments measure and track are described in the tables in Appendix 1. The instruments include:

1) A hospital facility survey (FS).

This survey instrument has been successfully used in previous work in Kenya [13] (a draft instrument is included in appendix 5) and will include a list of the staff responsible for admission clinical care and their qualifications (that will be updated as appropriate). Each clinician will be assigned a unique code used to label admission events for which they are responsible and only this code will be used in any analysis (see section on analysis). This code will be known only to research staff and the master file linking personal identifiers to this code will be destroyed at the end of the study in the interests of confidentiality. Surveys will be repeated 6-12 monthly (depending on group allocation) and as summarised in the table

below.

2) Health worker questionnaires, Focus group discussions and health worker skills assessments (HWQ).

i) At the time of each hospital survey health workers on duty (clinical, nursing and support staff) will be asked to complete confidential, self-administered questionnaires seeking their opinion on the level of provision of key services (n = 30 per hospital) as a supplement to the research team's facility survey (for example the adequacy of supply of oxygen). Verbal assent will be sought from health workers asked to complete these questionnaires that are based on previously used tools [13](see appendix 5).

ii) All medical, laboratory and nursing staff expected on duty for more than 2 days during a survey period (to allow collection and return of the questionnaire) will be asked to complete a self-administered, confidential questionnaire aimed at exploring their level of motivation and work satisfaction (HWSM). Three surveys will be completed at 12 monthly intervals (see appendix 5 for an example – work on the final instrument will be undertaken at the start of the study after recruitment of a Kenyan investigator and in collaboration with subject experts from South Africa).

iii) Complementing the questionnaire and supplementing the interview methods, the social scientist will conduct focus group discussions with selected staff from each hospital. 6-8 health workers will be recruited from discrete groups (nurses, doctors) for a one (1) hour discussion per hospital (8 hospitals in total). The discussions will centre on health workers' perspectives on their level of motivation and how it is influenced during and, in post-baseline discussions in intervention hospitals will explore how motivation is influenced by the hospital IMCI implementation. Considering that no intervention is planned to be implemented in the Faith-based hospitals, the discussions and interviews will focus on influences on motivation only.

iv) Specific hospital staff including senior medical and non-medical administrators and senior ward staff and DHMB members, after gaining their consent, will be interviewed at intervals during the study by a social scientist. The qualitative data collected will be used to build up an institutional diary of events and changes affecting the hospital that might affect its performance.

v) Some important clinical skills cannot be evaluated from retrospective review of case records. To evaluate the success and longevity of the training health workers providing front-line clinical care will be invited to take part in a skills re-assessment at each 12-

monthly survey (SS). Skills to be assessed in this way include the practice of triage, newborn and child resuscitation with the assessment making use of standardised clinical scenarios and video-recordings.

3. Retrospective review of hospital records (400 Rrev).

At the end of each 6-months (intervention) and 12-months (control) periods research staff undertaking the hospital survey will collect and record data (including outcome) from 400 admission episodes. These will be selected by randomly sampling calendar dates from the previous 6 (or 12) months and retrieving only records from children recorded as admitted on those dates in ward registers. The proportion of dates to be randomly selected will be estimated by dividing 400 by the number of admissions over the 6 (or 12) months period. If fewer than 30 records are retrieved for children with uncommon clinical conditions these will be supplemented by purposeful selection of cases identified from hospital discharge registers. No name or address data will be abstracted from these records and each will be provided with a unique study number for identification, rather than the hospital inpatient number, ensuring that patient data confidentiality is preserved.

4. Caretaker Interviews (CTI).

To supplement data from health worker questionnaires and record reviews the caretakers of children being discharged from hospital will be invited to complete a questionnaire (see appendix 5), during interview, if their child is discharged from hospital during the period of the 6 (or 12) monthly surveys (estimated 2 weeks survey period, permitting approximately 40 short interviews). Verbal consent will be sought for these interviews that will focus on the availability of drugs and investigations during the inpatient stay (did any need to be purchased), the caretaker's understanding of the condition resulting in admission and its discharge treatment (if any) and establishing the out of pocket costs to the family resulting from the admission. The draft tool is based on a previous successfully used instrument [14].

Surveys will be conducted over a period of 2 weeks by specifically trained project staff and will include as part of the teams the project paediatrician who will provide feedback and a member of staff from another, different intervention hospital. Major surveys will include data collection using the instruments FS, HWQ, 400Rrev and CTQ. The instruments SS and HWSM will be used on an annual basis only in both intervention and control hospitals. The timetable of data collection is summarised in the table.

Time period (months) from intervention	-3 to 0 m	1 to 3 m	3 to 5 m	6 to 8 m	9 to 11 m	12 to 14 m	15 to 17 m	18 to 20 m	21 to 23 m	24 to 26 m	27 to 30 m	31 to 33 m
**Intervention hospitals**												
Provision of guidelines, job aides, training		■	■									
Quality Imp Facilitation			■	■	■	■	■	■				
Major Survey	■			■		■		■		■		■
Motivation & HW skills	■					■				■		
Minor Survey / External supervision			■		■		■		■		■	
**Control hospitals**												
Provision of guidelines.		■										
Major surveys, written feedback	■					■				■		
**Independent surveys**	■								■			

ii) Data validation.

The survey team will be trained in survey methods for a period of at least 3 weeks at the beginning of the study including piloting of data collection tools in a facility that will not be part of the study. All pre-coded, categorical and quantitative variables will be entered in specific data entry programmes with in-built range and consistency checks. The on-site survey supervisor will be responsible for ensuring the quality of data collection and a random, 10% sample of the entries from selected case records will be cross checked on site to assess data quality. Qualitative data collected from interviews with senior staff in the hospital as part of the institutional diary will be transcribed and interviewees offered the opportunity to correct the transcription for errors of fact or meaning.

<u>Independent monitoring</u>. As the researchers are both implementers of the intervention and assessors of its success, the credibility of the study's results will be improved if consistent with the findings of an independent guarantor. The project will therefore provide for independent hospital assessments, co-ordinated with the Division of Child and Adolescent Health at WHO and using their 3-day assessment tool [12]. Group 1 and Group 2 hospitals will undergo these independent assessments within 3 months of their first survey and just before their 24-month survey. The results of these independent assessments will only be made known to the investigators at the end of the data collection period.

9) DATA MANAGEMENT.

Data will be collected as questionnaires and data abstraction forms and subsequently data entered into custom developed databases using Filemaker Pro in a PC format. Range and consistency checks will be built into the initial data entry. All databases will be collated and cleaned prior to analysis and final copies kept with the principal investigator on a PC with back-up copies on CDs. Individual level data will not include names or area of residence and all records will be indexed on and labelled by unique study record identifiers only. Only summary data and not individual level data will be provided to the hospitals in the form of feedback reports. Qualitative data will be stored as hard copy with the PI and abstracted to text format reports stored on a PC.

<u>Data Analysis (see tables 1 and 2 in appendix 1).</u>

For intervention hospitals 6 major surveys will define 6 periods of 6 months: pre-intervention (-5 to 0m), model intervention (1 to 6 m & 7 to 12m), model intervention & financial support to tackle key, non-consumable resource barriers (13 to 18m), continuing external supervision alone (19 to 24m), no active input (25 to 30m). For control hospitals 3 surveys will define 3 periods: pre-intervention (-5 to 0m) and after provision of guidelines and survey results (1 to 12 & 13 to 24m). The data collected will provide information relevant to domains (effectiveness, safety, timeliness and efficiency) included in the framework developed by the Institute of Medicine for assessing quality of care [39]. Where possible multiple approaches to assessment will be used (table 1, appendix 2) to permit 'triangulation' of findings as an aid to assessing their credibility.

Data representing 2.5-3.0 years from each hospital will therefore be used to describe changes in performance within hospitals between surveys and over the whole time period. The principal performance measures (see tables 1 & 2, appendix) were selected on the basis of one or more of: established public health importance; a clear, logical link to patient outcomes; a clear and proximate link to the intervention; favourable cost-effectiveness or requirement for minimal resource inputs; objectivity of the assessment(s).

<u>Primary outcome measures.</u>

Indicators that are most close related to the intervention rather than those at the end of potentially complex causal pathways where intervening variables or confounding threaten the plausibility of causal inference have been selected as primary outcome measures together with

effects on costs. Importantly these also most directly assess the degree to which the intervention is taken up.

Process measures will be evaluated in three ways:

1) Process performance indicators, with confidence intervals where appropriate, will be reported for each time period based on the 400 randomly selected records from that period. Assuming malaria, pneumonia and diarrhoea each represent at least 20% of cases (unpublished observation) 80 disease specific case records would be available for analysis. While optimally hospitals should be able to ensure appropriate, safe service provision at all times realistic aims for improvement in response to intervention are illustrated (table 2). Although the precision of survey estimates is modest where sample sizes are low (n = 30) for some indicators achievement of marked improvements may still credibly be attributed to an intervention effect rather than chance. As sample sizes increase changes as small as 10 percentage points observed between successive surveys may plausibly be associated with the intervention (see sample size section). These simple data will describe within hospital changes over time and thus any temporal association with the duration and nature of the intervention. If changes in the same direction and of the same magnitude are consistently observed across the sites and not observed in control hospitals this will increase the plausibility that the intervention is causing the effect and indicate that the effect is not site specific.

2) Specific support for the existence of a generalisable intervention effect will be sought by comparing changes in specific process measures (and other outcomes below) 24 months after intervention, including the correct use of first-line drugs in intervention and control hospitals, using exploratory, statistical models that take account of the hierarchical nature of the data and that permit adjustment for factors operating at the hospital and health worker levels.

3) Data from at least 20 case records will also be available to describe the routine assessment and treatment practices of at least 40 different health workers (at least 10 per hospital) for each year of observation. Linking this to data describing the health worker's pre-service training, their exposure to the intervention and measures of motivation we will be able to explore the effects of these and other factors on the degree to which admission care is provided in accordance with guidelines using generalised estimating equations [43].

The costs and cost-effectiveness of care for children in district hospitals. Costs and cost-effectiveness are key issues in the sustainability of any proposed new intervention, particularly one that seeks to justify additional investment in the health system. We intend therefore to assess the cost of the intervention per additional child receiving best practice admission care and the effect of the intervention on inpatient care costs. A full costing of the intervention programme, representing costs of training, job aides, supervision and local quality improvement activities, will therefore be undertaken by the economist employed as part of this fellowship and supervised by Dr. Kara Hanson from LSHTM. The average costs of inpatient care, taking the provider and household perspectives, will be derived. It is anticipated that data collection on resource use (diagnostic tests, drugs, consumables etc) for 120 children admitted with malaria, pneumonia, diarrhoea, neonatal sepsis and malnutrition will be available from caretaker questionnaires conducted during the surveys and the costs pre-intervention and in the second year of the intervention in all eight hospitals will be estimated. Sub-analyses will explore whether there are differences in treatment costs when care is actually provided according to best practice standards or not.

<u>Secondary or general outcome measures.</u>

These are defined as outcome measures that are potentially affected by factors beyond the control of the project team (eg. a major change in the economy, a respiratory disease epidemic). Thus while it is important that they are examined attributing changes in the indictors to the intervention must be very cautious, with interpretation taking account of additional, carefully documented potential explanations.

Mortality and cause specific case fatality rates will be described for each 6 months (intervention) and 12 months (control) periods.

The care environment and resources both capital and consumable resources, and the organisation of care will be assessed against key defined standards (table 2, appendix) at each time point.

10) TIME FRAME:

Project Staff recruitment, final tool developments and training	-	October 2005– April 2006.
Conduct of intervention study	-	May 2006 to October 2009
Final, analysis and report preparation	-	October 2009 – September 2010.

11) ETHICAL CONSIDERATIONS:

- This intervention study proposal has been developed with the Ministry of Health to test a strategy for improving paediatric hospital care in line with a national programme to implement IMCI.
- Hospitals will be selected and allocated to intervention or control groups on the basis of pre-defined criteria.
- Control hospitals will receive copies of paediatric care guidelines and job aides and formal, constructive feedback from 3 detailed, annual surveys from which they may gain benefit. Intervention hospitals will receive a more complete package of supervision and support for local quality improvement activities.
- The study will not prevent control (or intervention) hospitals from receiving new assistance, staff, or other interventions delivered by governmental or non-governmental organizations during the study period. The aim is to collect information on changes that may be occurring in the health sector independent of the study-related interventions, these include the implementation of new policies or programmes.
- No procedures other than training and feedback for hospital staff, retrospective review of case records, observation of health care delivery and use of questionnaires will be employed in this study. After providing the facility and the health workers with information about the study verbal assent will be requested to continue with self-administered questionnaires while written consent will be requested for face to face interviews. Individuals will be offered the opportunity to refuse an interview without prejudice. Anonymous self-administered questionnaires will be used to assess motivation and there will be no follow up for non-responders.
- In particular it will be explained to health workers that an overall picture of care and health worker skills is the aim, that an individuals performance is not being judged and that the aim is not to highlight or punish an individual's "poor performance". Instead the aim is to improve the overall performance of the hospital in delivering paediatric care.
- As the study will take almost 3 years the process of dialogue with hospital staff will be an ongoing and participatory activity with staff being invited to join the research team in a number of local research / problem solving activities.
- Feedback on the progress of the study and the effect of the intervention is again part of the participatory study design. The specific aim is to engage local staff and the DHMB in

addressing local issues that will improve the care of children coming to hospital.

- It is a specific aspect of this interactive research that practices considered dangerous or inadequate will be brought to the attention of hospital staff although in a non-threatening, constructive manner. Where a member of the research team is present at the time that dangerous care is being given, and she/he is suitably qualified to intervene, the safety of the patient is their first priority. Ideally any specific intervention will be after discussion with the most senior member of the project team available and with senior local hospital staff.
- All records will be assigned a unique record number that will be used for data storage and analysis. Data abstracted from hospital records and stored for analysis will not include a child's name and hospital number. Health workers responsible for admissions will be assigned a unique identifying code known only to specific research staff. The link between this code and the health worker's name will be destroyed prior to finalising the database and before final analysis. Health workers names or unique identifiers will never be used in any feedback or reports.
- Data will be kept securely with password protected access for research staff only.
- An improved quality of care is clearly desirable for all children admitted to Kenyan hospitals. It is hoped that intervention and to some degree control hospitals and the children they care for will benefit directly from inclusion in the study. It is hoped that this project will also contribute to the Government of Kenya's overall health objectives of improving the quality of care by providing important information on one means of achieving this in district hospitals.
- The conduct of this study is expected to provide 1 paediatrician, 1 economist and 1 social scientist from Kenya with the opportunity to undertake PhDs.

Animal Subjects.

Not applicable

12) EXPECTED APPLICATION OF THE RESULTS.

Hospitals taking part in the survey will be given direct and ongoing feedback in a variety of formats: individualised written reports (control and intervention hospitals), feedback visits (for staff and the DHMB), intermittent supervision and local quality assurance meetings (intervention hospitals). It is hoped the data will therefore directly influence patient care.

Reports (but not individual level data) will also be shared freely with the Ministry of Health and Kenya’s two medical schools (all collaborators on this proposal) and with the WHO internationally. It is hoped that this will facilitate their efforts to improve quality of care in the Kenyan health system with valuable lessons for other countries in the region. It is anticipated that the results will also be presented locally and internationally and published by a group of largely Kenyan research staff to develop local research capacity and provide a wider audience for important findings.

13) REFERENCES.

1. Kenya Demographic and Health Survey 2003 - Preliminary Report. Nairobi: Central Bureau of Statistics, Nairobi, Kenya., 2003.
2. Commission on Macroeconomics and Health - Chair S, JD. Macroeconomics and Health: Investing in Health for Economic Development. Geneva: WHO, 2001.
3. Lee J. Child survival: a global health challenge. *Lancet* 2003;**362:**262.
4. Bryce J, el Arifeen S, Pariyo G, Lanata C, Gwatkin D, Habicht J-P. Reducing child mortality: can public health deliver? *Lancet* 2003;**362:**159-164.
5. Jones G, Steketee R, Black R, Bhutta Z, Morris S, Group BS. How many child deaths can we prevent this year? *Lancet* 2003;**362:**65-71.
6. The Bellagio Study Group for Child Survival. Knowledge into action for child survival. *Lancet* 2003;**362:**323-327.
7. English M, Lanata C, Ngugi I, Smith P. The District Hospital. In: Jamison D, Alleyne G, Breman J, et al., eds. Disease Control Priorities in Developing Countries - 2nd Edition. Washington DC: World Bank, 2004.
8. Anonymous. Mexico, 2004: GLobal Health needs a new research agenda. *The Lancet* 2004;**364:**1555-1556.
9. Lavis J, Posada F, Haines A, Osei E. Use of research to inform public policy making. *The Lancet* 2004;**364:**1615-1621.
10. Simoes E, Peterson S, Gamatie Y, Kisanga F, Mukasa G, Nsugwa-Sabiti J, Were W, Weber M. Management of severely ill children at first-referral lelvel facilities in sub-Saharan Africa when referrral is difficult. *Bulletin of the World Health Organisation* 2004;**81:**522-531.
11. Snow R, Mungala V, Forster D, Marsh K. The role of the district hospital in child survival at the Kenyan Coast. *Afr J Health Sci* 1994;**1:**11-15.
12. Nolan T, Angos P, Cunha A, Muhe L, Qazi S, Simoes EA, Tamburlini G, Weber M, NF P. Quality of hospital care for seriously ill children in less-developed countries. *The Lancet* 2000;**357:**106-110.
13. English M, Esamai F, Were F, Wasunna A, Ogutu B, Peshu N, Snow R. Assessment of inpatient paediatric care in first referral level hospitals in 13 districts in Kenya. *The Lancet* 2004;**363:**1948-1963.
14. English M, Esamai F, Were F, Wasunna A, Ogutu B, Peshu N, Snow R. Delivery of paediatric care at the first referral level in Kenya. *The Lancet* 2004;**324:**1622-1629.
15. Gwatkin D, Bhuyia A, Victora C. Making health systems more equitable. *The Lancet* 2004;**364:**1273-1280.
16. United Nations Development Programme. Millenium Development Goals., 2003 http://www.undp.org/mdg/.
17. Noor A, Gikandi P, Hay S, Muga R, Snow R. Creating spatially defined databases for equitable health service planning in low-income countries: the example of Kenya. *Acta Tropica* 2004;**91:**239-251.
18. Ibrahim JE. Performance indicators from all perspectives. *Int J Qual Health Care* 2001;**13**(6)**:**431-432.
19. Owino W, Korir J. Public Health Sector Efficiency in Kenya: Estimation and Policy Implications. Nairobi: Institute of Policy Analysis and Research., 1997.
20. Berkley J, Lowe B, Mwangi I, Williams T, Bauni E, Mwarumba S, Ngetsa C, Hart A, Maitland K, English M, Scott J, Marsh K. Community acquired bacteremia amongst children admitted to a rural district hospital in Kenya. *NEJM* 2005; 352: 39-47.
21. Marsh K, Forster D, Waruiru C, Mwangi I, Winstanley M, Marsh V, Newton C, Winstanley P, Warn P, Peshu N, Pasvol G, Snow R. Indicators of life-threatening malaria in African children. *New England Journal of Medicine* 1995;**332:**1399-1404.

22. World Health Organisation. Management of the child with a serious infection or severe malnutrition. Geneva: World Health Organisation, 2000.
23. Oliveira-Cruz V, Hanson K, Mills A. Approaches to overcoming health system constraints at the preipheral level: review of the evidence: Commission on Macroeconomics and Health, 2001.
24. Blaauw D, Gilson L, Penn-Kekana L, Schneider H. Organisational relationships and the 'software' of health sector reform. Disease Control Priorities Project Background Paper. Washington, DC., 2003.
25. Barnum H, Kutzin J. Public Hospitals in Developing Countries. Baltimore: The Johns Hopkins University Press, 1993.
26. Franco L, Bennett S, Kanfer R, Stubblebine P. Determinants and consequences of health worker motivation in hospitals in Jorda and Georgia. *Social Science & Medicine* 2003;**58:**343-355.
27. Hongoro C, McPake B. How to bridge the gap in human resources for health. *The Lancet* 2004;**364:**1451-1456.
28. Lanier D, Roland M, Burstin H, Knottnerus J. Doctor performance and public accountabiltiy. *Lancet* 2003;**362:**1404-1408.
29. Grol R, Grimshaw J. From best evidence to best practice: effective implementation of change in patients' care. *The Lancet* 2003;**362:**1225-1230.
30. O'Brien T, Freemantle N, Oxman A, Wolf F, Davis D, Herrin J. Continuing education meetings and workshops: effects on professinal practice and health care outcomes. *The Cochrane Library* 2002;**Issue 4.**
31. O'Brien T, Oxman A, Davis D, Haynes R, Freemantle N, Harvey E. Audit and feedback: effects on professional practice and health care outcomes. *The Cochrane Library* 2002;**Issue 4.**
32. Massoud R, Askov K, Reinke J, Franco L, Bornstein T, Knebel E, MacAulay C. A Modern Paradigm for Improving Healthcare Quality. Bethesda: Quality Assurance Project, 2001.
33. Duke T, Tamburlini G. Improving the quality of paediatric care in peripheral hospitals in developing countries. *Arch Dis Child* 2003;**88:**563-565.
34. Lilford R, Mohammed M, Spiegelhalter D, Thompson R. Use and misuse of process and outcome data in managing performance of acute medical care: avoiding institutional stigma. *The Lancet* 2004;**363:**1147-54.
35. Rubin H, Provonost P, Diette G. The advantages and disadvantages of process based measures of health care quality. *Int J Qual Health Care* 2001;**13:**469-474.
36. Parry GJ, Gould CR, McCabe CJ, Tarnow-Mordi WO. Annual league tables of mortality in neonatal intensive care units: longitudinal study. *BMJ* 1998;**316**(7149)**:**1931-1935.
37. Haines A, Cassels A. Can the millennium development goals be attained? *BMJ* 2004;**329**(7462)**:**394-397.
38. Loeb JM. The current state of performance measurement in health care. *Int J Qual Health Care* 2004;**16**(suppl_1)**:**i5-9.
39. Envisioning the National Health Care Quality Report. Washington, DC.: Institute of Medicine., 2001.
40. Drummond MF, Jefferson TO. Guidelines for authors and peer reviewers of economic submissions to the BMJ. *BMJ* 1996;**313**(7052)**:**275-283.
41. Tamburlini G, Di Mario S, Maggi R, Vilarim J, Gove S. Evaluation of guidelines for emergency triage assessment and treatment in developing countries. *Arch Dis Child* 1999**:**478-482.
42. English M, Esamai F, Were F, Wasunna A, Ogutu B, Peshu N, Snow R. A survey of inpatient paediatric care provided by first referral level hospitals in Kenya - Final Report. Kilifi: Wellcome Trust / KEMRI Collaborative Research Programme., 2003.

43. Zurovac D, Rowe A, Ochola S, Midia B, English M, Snow R. Predictors of the quality of health worker treatment practices for uncomplicated malaria at governemnt health facilites in Kenya. *International Journal of Epidemiology* 2004;**33:**1080-1091.
44. Gibberd R, Hancock S, Howley P, Richards K. Using indicators to quantify the potential to improve the quality of health care. *Int J Qual Health Care* 2004;**16**(suppl_1)**:**i37-43.

Appendix 1.

A basic framework illustrating the complexity of actors, the centrality of health workers and the major interactions relevant to the provision of the proposed intervention to improve the quality of care.

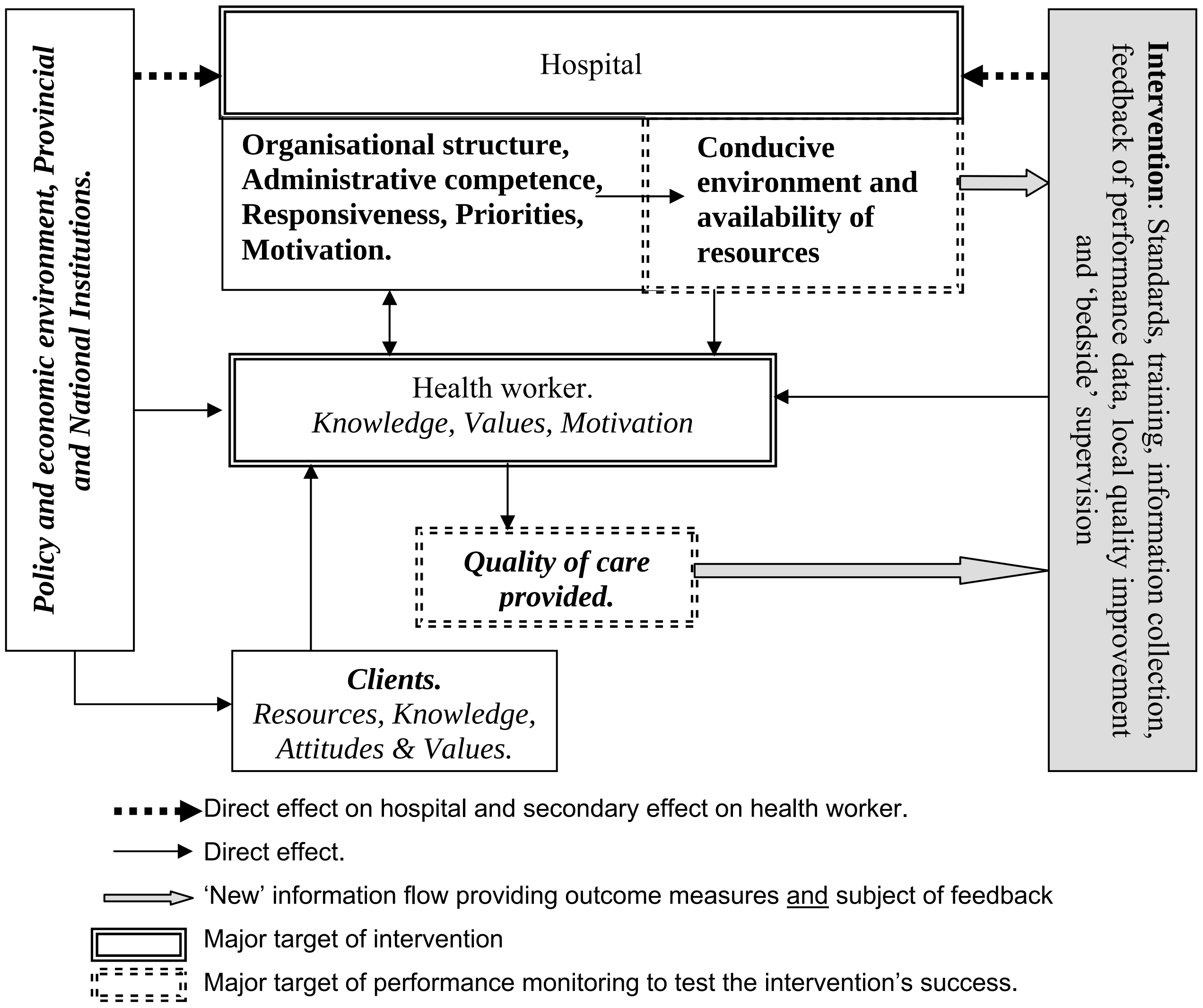

Major performance indicators and means of assessment.

Area of Assessment		Intervention Group	Control Group
Environment & Capital Resources			
Hand-washing		Obs 3m / FS / HWQ	Obs 12m / FS / HWQ
Ward hygiene		Obs 3m / FS / HWQ	Obs 12m / FS / HWQ
Nursery equipment		Obs 3m / FS / HWQ	Obs 12m / FS / HWQ
Consumable resources			
Oxygen		Obs 3m / FS / HWQ	Obs 12m / FS / HWQ
First line drugs		Obs 3m / FS / HWQ / CTI	Obs 12m / FS / HWQ / CTI
Second line drugs		Obs 3m / FS / HWQ	Obs 12m / FS / HWQ
Organisation of Care			
Triage		Obs 3m	Obs 12m
Emergency care		Obs 3m	Obs 12m
Ward prioritisation		Obs 3m	Obs 12m
Nutrition		Obs 3m / FS / HWQ / CTI	Obs 12m / FS / HWQ / CTI
Blood transfusion		Obs 3m / HWQ / 30 RRev	Obs 12m / HWQ / 30 RRev
Correct assessment and treatment			
Malaria<br>Pneumonia<br>Diarrhoea / dehydration	Clinical classification<br>Drug(s) chosen<br>Drug doses	400 RRev	400 RRev
Newborn Care<br>Severe Malnutrition	Drug(s) chosen<br>Drug doses	30 RRev	30 RRev
HIV	Cases screened<br>Septrin prophylaxis	400 RRev	400 RRev
Lumbar puncture	Number / indications	400 RRev	400 RRev
Skills assessment		SS (annual)	SS (annual)
Policy implementation			
Missed vaccines		400 RRev / CTI	400 RRev / CTI
Vitamin K at birth		30 RRev	30 RRev
Vitamin A at admission		400 RRev	400 RRev
Ensuring discharge treatment understood		CTI	CTI
Caretakers and Health Workers			
Caretaker knowledge:	Condition<br>Treatment<br>Discharge drugs	CTI	CTI
Health worker motivation		HWQ	HWQ

Obs = Direct Observation, 3m, 6m, 12m = 3,6 & 12 monthly respectively, FS = Facility Survey, CTI = Caretaker Interview, 30 RRev = Record review of 30 cases, 400 RRev = Record review of 400 cases, SS = skill station, HWQ = Health Worker Questionnaire

Standards required and expected scale of Intervention Effect.

Area of Assessment	Previous Data	Desired Standards	
**Environment, Capital and Consumable Resources, Organisation and Quality of Care**			
Hand-washing	In 14/14 hospitals water, sinks and soap were mostly available, practices were not examined.	Clean sink, water and soap available 100% of time and staff wash hands between handling every nursery and diarrhoea admission.	
Ward hygiene	In 12/14 hospitals 20% or more of caretakers and in 11/14 hospitals half of staff felt patient bathrooms were inadequate.	Clean, functional washing facilities and toilets for patients and caretakers, separation of diarrhoea cases and adequate facility for isolation	
Nursery equipment	11/14 hospitals had some equipment to warm infants, though sharing incubators was very common, 6/14 hospitals rarely or never had phototherapy equipment.	Babies < 1.75kg can be kept warm, no baby has to share a cot, phototherapy and oxygen available for distressed newborns when required.	
Oxygen	58% children prescribed oxygen received it, need is likely to have been underestimated	Oxygen is available for all children that need it	
Triage	Formal triage was not present in any of 14 hospitals.	Effective triage operational	
Emergency care	Newborn ambu-bags were not available in 4/14 hospitals, skills were not assessed.	Equipment for emergency care available in maternity, ward and outpatients.	
Ward prioritisation	Formal prioritisation of ward care was not observed in any of 14 hosptials.	Effective prioritisation operational	
Quality of malaria blood slide	No data, but health workers commonly report that they do not believe results.	Hospital result >80% sensitive and specific compared with gold standard	
Quality of CSF microscopy	Lumbar puncture rarely performed, although 13/14 hospitals said it could be done	Equipment available and staff competent to undertake CSF microscopy, service available at least 8am to 4pm.	
**Assessment, treatment, practice and drug availability**		**Base Estimate**	**Post Intervention**
All acute medical paediatric admissions	<20% of cases of malaria, pneumonia and diarrhoea/dehydration were both adequately documented and received treatment according to guidelines.	<20% adherent to guidelines	60% adherent to guidelines
First line drugs	In 12/14 hospitals essential items were reported present ≥ 66% time	Available 66% of occasions	Available 90% of occasions
Second line drugs	In ≥ 11/14 hospitals Cloxacillin & ceftriaxone / cefotaxime were rarely or never available.	Available 20% of occasions	Available 60% of occasions
Nutrition	11/14 hospitals rarely or never had appropriate feeds for malnourished children or newborn formula, Vitamin A was given to 33%, mineral supplements to none.	<20% malnourished children receive adequate nutrition	60% adherent to guidelines
Blood transfusion	40% of blood transfusions did not appear to be warranted, no data is available on delivery in <4hours for those with severe anaemia and respiratory distress.	40% transfusions unwarranted	< 10% unwarranted, 50% within 4 hours for urgent cases
Diagnosis of HIV	Diagnosis of HIV was very rare, the number eligible for testing is not known.	HIV ignored	HIV considered and testing promoted in >60% appr. cases
Drug errors and use	Dosage errors occur >10% of occasions, >50% diarrhoea cases receive antibiotics, over-use of iv quinine and treatment for very severe pneumonia is likely to be common.	Dose errors 10%<br>Inappropriate drug/dose 40%	Dose errors <3%<br>Inappropriate drug/dose 10%
Basic resuscitation	No data, personal observation is that <30% staff can handle a bag and mask effectively	<8/20 adequately skilled	16/20 adequately skilled
**Policy implementation**			
Missed vaccines	<10% inpatients less than 1 year old have their immunisation status checked.	Immunisation checked <10%	Immunisation checked 60%
Vitamin K at birth	In ≥ 11/14 hospitals Vitamin K is not given.	<20% births given Vit K	80% births given Vit K
Vitamin A at admission	<10% admissions without malnutrition receive Vitamin A	<20% admissions given Vit A	60% admissions given Vit A
Ensuring discharge treatment understood	In 9/14 hospitals < 25% caretakers knew how often to administer discharge drugs.	<25% caretakers understand discharge drugs	60% caretakers understand discharge drugs

Appendix 2. Study Introduction / Information.

A study to examine an approach to implementing Referral Care – IMCI – An introduction for participating health workers.

The Kenyan Ministry of Health aims to provide "sustainable, quality health care that is acceptable and affordable". Improving the performance of the health service and reducing child mortality from key diseases (malaria, measles, pneumonia, diarrhoea, problems in the newborn period) and malnutrition are specific target areas. Improving inpatient care, particularly for these common causes of inpatient mortality in children may be assisted by the introduction of evidence-based practice guidelines and addressing problems of service delivery. Practice guidelines for district hospitals have been adapted from WHO recommendations by the Ministry of Health as part of their effort to implement the Integrated Management of Childhood Illnesses approach. KEMRI / Wellcome Trust, the Ministry of Health and other partners are testing ways of providing these guidelines to district hospitals to see how best to help health workers develop new skills and help hospitals to identify ways to improve the care they offer to children.

We are inviting you and your hospital to take part in this research. In total we will be working with eight hospitals. The main Kenyan groups involved in this research include:

KEMRI / Wellcome Trust
The Ministry of Health,
The University of Nairobi
Moi Teaching and Referral Hospital

[For intervention hospitals]

What will the research involve?
The research team will begin by undertaking a survey of how care is provided now. This will involve looking at hospital records, seeing what resources are available, asking some health workers to fill questionnaires and conducting interviews with hospital staff and children's caretakers. We will only collect information from people willing to give it – we will need your permission and will explain this to people in more detail when appropriate.

After the first survey we will give you and the hospital the findings and arrange for hospital staff to receive training on the new guidelines.

After the training we would like to work with the hospital to tackle issues that make it difficult to provide the correct forms of care for children. We hope that many health workers will become involved in these local activities.

Every six months a new survey will be conducted to see whether the care offered to children has changed. We will bring the results of this survey back to you and explain it and every 3 months the paediatrician from the research team will come and visit and offer support.

How long will the research take?
We hope to work directly with the hospital to tackle the problems with paediatric care for 18 months – during this time we hope some health workers will have learned how to continue with the process themselves.

We will conduct 5 surveys after the initial survey over a total period of 2.5 yrs to see whether providing the training and support makes any long-term benefit to the hospital.

[For control hospitals]

What will the research involve?
The research team will begin by undertaking a survey of how care is provided now. This will involve looking at hospital records, seeing what resources are available, asking some health workers to fill questionnaires and conducting interviews with hospital staff and children's caretakers. We will only collect information from people willing to give it – we will need your permission and will explain this to people in more detail when appropriate.

After the first survey we will give you and the hospital the findings and arrange for hospital staff to receive the new guidelines for care of children.

We will repeat the survey on two further occasions at 12 months intervals to see whether the care offered to children has changed. We will give the results of this survey back to you and explain them.

These repeat surveys are to see whether providing the guidelines and results of surveys are of any long-term benefit to the hospital and its patients.

What is done if there is poor performance?
The research will never identify and comment an individual's performance. This process is not about seeing who is to blame or finding out who is good or bad. Its aim is to consider how the hospital is performing as a team and how the team can be helped to improve the care given to sick children. The process of finding out what the problems are is so that we can all try to solve them, not to decide who to blame for them.

What will the research achieve?
We hope to improve understanding of how to improve the care for children in hospital by learning with and from you. We hope your hospital will get some direct benefit from the research activities and results. If we can find a way to improve care that works then it may help improve care for children all over Kenya.

Thank you.

Appendix 3. – Consent explanation sheets for health worker and caretaker interviews.

A study to examine an approach to implementing Referral Care – IMCI

Consent Information Sheet for Interviews with health workers.

About KEMRI

KEMRI is part of the Ministry of Health and runs research activities to find out more about illness and how to manage illness in Kenya. The overall aim is to improve health and well being for people in Kenya and other parts of Africa.

In the research at this hospital KEMRI is working with other Kenyan groups, the main ones being The Ministry of Health, The University of Nairobi and Moi Teaching and Referral Hospital

What is this research activity?
We are examining ways of improving the care given to children who come to hospital. Working with the Ministry of Health guidelines have been developed to help health workers give the best, most appropriate care for sick children given the resources that are commonly available. Our research is trying to find out how easy or difficult it is for health workers to follow this advice. We are doing this by providing the guidelines (intervention sites - and training and supervision) and examining the way care for children is given in regular surveys. One important way of seeing what types of problems there are when trying to improve care is to ask people what works well and what doesn't work in their hospital. We would like to ask you some questions to get your opinion on what works and what doesn't and why.

Who are we approaching?
We are approaching a wide range of people to help us with the interviews. Health workers of all types, hospital administrators and members of the hospital management board.

What are we asking people to do?
We would like to ask you some questions and record your answers.

☐ Agreeing to be interviewed. This will involve:

a. Giving up 20 - 30 minutes of your time so I can gather your views on the way in which children are cared for at this hospital, what resources are available and what could be improved.
b. Agreeing that we can record your comments (see consent sheet).
c. Allowing us to use what you say in the interview, together with the comments of others, to build up a picture of how well things work at the hospital and what needs to change to make things work better.

(For those to be repeatedly interviewed as part of an institutional diary)

d. Allowing us to come back to you in the future to ask you similar questions to see whether anything is changing.

Confidentiality.
Your name and job title will not be used in any reports of this work. Only a code number will appear on the record made of the interview(s) and only the research team will have access to the link between the code numbers and individuals. Only general terms will be used to indicate who

took part such as: senior staff in the hospital. We will offer you the opportunity to change the record of the interview until you are happy with it if you would like. No one other than the research team and yourself will be allowed to see the record of the interview without your permission.

Risks of the research.
We do not believe there are any risks to taking part in this research.

Benefits of the research

We are unable to offer any individual benefits for participating in this research. However it is hoped that understanding why things go well / poorly with attempts to improve care for children in the hospital will help define better ways of improving hospital care in the future.

Voluntary Participation.
There is no obligation at all to help with this study and there will be no penalties of any kind if you decide not to be interviewed. If you do agree to provide information for this study at any time you may either terminate the interview or contact us later and ask for all records of the interview to be destroyed, again without penalty.
(For those to be repeatedly interviewed as part of an institutional diary)
If you agree to be interviewed to day it does not commit you to any interviews in the future. Any time we would like to interview you at a later date we will seek your permission and ask for your verbal consent and you will be free to refuse an interview without it causing any trouble.

Do you have any questions?

<u>Consent Agreement for Health Workers for study to examine an approach to implementing Referral Care – IMCI.</u>

I, ____________________________________have been informed about the study entitled:

Assessing the impact of an intervention to implement the referral care component of Integrated Management of Childhood Illnesses in district hospitals.

under the direction of Dr. M. English and have been provided with information concerning this study to help me understand it. The implications, duration, purpose, voluntary nature and inconveniences or risks that may reasonably be expected have been explained to me by:

____________________________*(name of person taking consent).*

I have been given the opportunity to ask questions concerning the study and these have been answered to my satisfaction. If I have further questions, I may contact:

Dr. M. English
P.O. Box 43640
Tel.2720163
Nairobi.

I understand that I may at any time during the study revoke my consent without any loss or penalty and that the information I have contributed will then be destroyed.

I confirm that I:

1) Am happy to be interviewed
2) Am / Am not* happy for a tape recording of the interview to be made in addition to written notes (if recording device available).

* *Delete as appropriate.*

Signed:____________________________ Date ____ / ____ / __________

Signature of person taking consent

____

Appendix – Consent explanation sheet for health worker and caretaker interviews.

A study to examine an approach to implementing Referral Care – IMCI

Consent Information Sheet for focus group discussions with health workers.

About KEMRI

KEMRI is part of the Ministry of Health and runs research activities to find out more about illness and how to manage illness in Kenya. The overall aim is to improve health and well being for people in Kenya and other parts of Africa.

In the research at this hospital, KEMRI is working with other Kenyan groups, the main ones being The Ministry of Health, The University of Nairobi and Moi Teaching and Referral Hospital

What is this research activity?
We are examining ways of improving the care given to children who come to hospital. Working with the Ministry of Health guidelines have been developed to help health workers give the best, most appropriate care for sick children given the resources that are commonly available. Our research is trying to find out how easy or difficult it is for health workers to follow this advice. We are doing this by providing the guidelines (intervention sites - and training and supervision) and examining the way care for children is given in regular surveys. One important way of seeing what types of problems there are when trying to improve care is to ask people what works well and what doesn't work in their hospital. One of the things that people have suggested may affect the way services are provided is the degree of motivation of health workers. We would like to ask you some questions to get your opinion on what works and what doesn't in this hospital and why and in particular what affects health workers motivation.

Who are we approaching?
We are approaching a wide range of people to help us with the interviews. Health workers of all types at this hospital will be invited to take part.

What are we asking people to do?
We would like to ask some questions of you and others as a group and record the answers.

□ Agreeing to participate in the focus group discussion. This will involve:

e. Giving up 40 - 60 minutes of your time so I can gather your views on the way in which children are cared for at this hospital and what affects the staff's motivation.
f. Agreeing that we can record your comments (see consent sheet).
g. Allowing us to use what you say in the discussion, together with the comments of others, to build up a picture of how well things work at the hospital, what affects motivation and what needs to change to make things work better.

Confidentiality.
Your name and job title will not be used in any reports of this work. Only a code number will appear on the record made of the interview(s) and only the research team will have access to the link between the code numbers and individuals. Only general terms will be used to indicate who took part such as: clinical staff in the hospital. We will not report a comment made by you in a way that will allow people to identify you.

Risks of the research.
We do not believe there are any risks to taking part in this research.

Benefits of the research

We are unable to offer any individual benefits to you for participating in this research. However it is hoped that understanding why things go well / poorly with attempts to improve care for children in the hospital will help define better ways of improving hospital care in the future.

Voluntary Participation.
There is no obligation at all to help with this study and there will be no penalties of any kind if you decide not to join the group discussion. If you do agree to join the discussion group at any time you may either leave the discussion or contact us later and ask for all the information you provided to be destroyed, again without penalty.

Do you have any questions?

<u>Group Consent Agreement for Health Workers for study to examine an approach to implementing Referral Care – IMCI and exploring motivation.</u>

I have been informed about the study entitled:

Assessing the impact of an intervention to implement the referral care component of Integrated Management of Childhood Illnesses in district hospitals.

under the direction of Dr. M. English and have been provided with information concerning this study to help me understand it. The implications, duration, purpose, voluntary nature and inconveniences or risks that may reasonably be expected from taking part in the focus group discussion have been explained to me by:

_______________________________*(name of person taking consent).*

I have been given the opportunity to ask questions concerning the study and these have been answered to our satisfaction. If we have further questions, we may contact:

Dr. M. English
P.O. Box 43640
Tel.2720163
Nairobi.

I understand that we may at any time during the study revoke our consent without any loss or penalty and that the information we have contributed will then be destroyed.

Group consent

Are you willing to participate in this group discussion? **Y/N** /___/

If you agree to participate you are requested to sign in the space below as a sign of having understood the purpose and content of the discussion.

I _____________________________________ confirm that sufficient information regarding the discussion has been given and that I have willingly accepted to take part in this discussion.

Signature ______________________ Date: ______________________________

Name of witness ______________________ Signature ___________________________

Date: _________________________________

A study to examine an approach to implementing Referral Care – IMCI

Consent Information Sheet for Interviews with Caretakers of admitted children.

<u>About KEMRI</u>

KEMRI is part of the Ministry of Health and runs research activities to find out more about illness and how to manage illness in Kenya. The overall aim is to improve health and well being for people in Kenya and other parts of Africa.

In the research at this hospital KEMRI is working with other Kenyan groups, the main ones being The Ministry of Health, The University of Nairobi and Moi Teaching and Referral Hospital.

The research team is not part of the hospital but is trying to help the hospital improve its care of children.

<u>What is this research activity?</u>
We are examining ways of improving the care given to children who come to hospital. Working with the Ministry of Health guidelines have been developed to help health workers give the best, most appropriate care for sick children given the resources that are commonly available. Our research is trying to find out how easy or difficult it is for health workers to follow this advice. We are doing this by providing the guidelines (intervention sites - and training and supervision) and examining the way care for children is given in regular surveys. One important way of assessing the care given to children is to look at the experiences of people using the hospital. We are particularly interested in what types of care were offered, whether things needed to give good care were available, whether your child's problem and any remaining treatment was explained to you and what it has cost you and your family to obtain this care. We would like to ask you some questions about these issues so that we can understand how the hospital can improve and be better able to help sick children.

<u>Who are we approaching?</u>
We are approaching the parents / guardians / caretakers of children who are being discharged from hospital while the research team is here.

<u>What are we asking people to do?</u>
We would like to ask you some questions and record your answers.

□ Agreeing to be interviewed. This will involve:

h. Giving up 20 - 30 minutes of your time so I ask you the questions.
i. Agreeing that we can record your comments (see consent sheet).
j. Allowing us to use your answers, together with those of others, to build up a picture of how well things work at the hospital and what needs to change to make things work better.

<u>Confidentiality.</u>
Your name and your child's name will not be used in any reports of this work. Only a code number will appear on the record made of the interview(s) and only the research team will have access to the link between the code numbers and individuals. When we make a report we will only say things like: 'some caretakers said….' or '6 out of 10 caretakers reported….'.

Risks of the research.
We do not believe there any risks to taking part in this research.

Benefits of the research

We are unable to offer any individual benefits for participating in this research. However it is hoped that understanding why things go well / poorly with attempts to improve care for children in the hospital will help define better ways of improving hospital care in the future.

Voluntary Participation.
There is no obligation at all to help with this study and there will be no penalties of any kind if you decide not to be interviewed. If you do agree to provide information for this study at any time you may terminate the interview without penalty.

If you agree to be interviewed to day it does not commit you to helping the research team in any other way.

Do you have any questions?

Consent Agreement for Caretakers for study to examine an approach to implementing Referral Care – IMCI.

I, ______________________________________have been informed about the study entitled:

Assessing the impact of an intervention to implement the referral care component of Integrated Management of Childhood Illnesses in district hospitals.

under the direction of Dr. M. English and have been provided with information concerning this study to help me understand it. The implications, duration, purpose, voluntary nature and inconveniences or risks that may reasonably be expected have been explained to me by:

______________________________*(name of person taking consent).*

I have been given the opportunity to ask questions concerning the study and these have been answered to my satisfaction. If I have further questions, I may contact:

Dr. M. English
P.O. Box 43640
Tel.2720163
Nairobi.

I understand that I may at any time during the interview revoke my consent without any loss or penalty and that the information I have contributed will then be destroyed.

I confirm that I:

3) Am / Am not* happy to be interviewed

* *Delete as appropriate.*

Signed:_____________________________ Date ____ / ____ / __________

Signature of person taking consent

____

Appendix 4. Role of Investigators and Collaborator CVs.

Dr. Mike English	Responsible for co-ordinating proposal development, liaison with the Ministry of Health and collaborators, supervising training and data collection, data analysis and report preparation.
Dr. A. Wamae,<br>Dr. P. Migiro,<br>Prof. F. Esamai,<br>Prof. A. Wasunna,<br>Dr. F. Were,<br>Dr. B. Ogutu,<br>Prof. R.W. Snow,<br>Dr. N. Peshu.	Responsible for proposal development, monitoring of the survey team, membership of project steering group, interpretation and analysis of data and report writing.
Dr. G. Fegan<br>Dr. A. Rowe	Responsible for proposal development, data monitoring, statistical analysis and interpretation and report writing.
Prof. L. Gilson	Responsible for supervising the development of questionnaires to explore health worker satisfaction and motivation, supervisory support to Kenyan social scientist and analysis of data and report writing
Dr. K. Hanson	Responsible for supervising the development of models for cost evaluation and cost-effectiveness analysis, supervisory support to Kenyan economist and analysis of data and report writing.
Dr. M. Weber	Liaison person within WHO Geneva and responsible for organising independent monitoring of hospital performance through WHO.

Appendix 5. Draft Questionnaires.

Initial pilot work will inform revision of these questionnaires. The current questionnaires are based on those used in the 2002 KEMRI SSC approved study # 680 unless otherwise stated Included forms:

1) Draft Health Facility Survey
2) Draft Health Worker Questionnaire
3) Draft Caretaker Questionnaire
4) Copy of South African Questionnaire for assessing staff satisfaction / motivation that will be developed for the Kenyan context as part of this study.

Facility Survey

Hospital: ________________________________ HN | |

Information Provided by (Title of post)	Date

Introduction:
The purpose is to describe the resources available to the facility to assist in understanding what it is actually possible for the facility to achieve.

Part A
Hospital information and constraints

1. General information:

Number of beds in the paediatric department/ ward?		beds
Number of cots for neonatal / young infant admissions?		cots
Annual number of deliveries at the hospital?		per year

What is the **actual** (average) number of staff at the hospital and in the paediatric ward(s):

	Hospital (all dept.)	Paediatric dept/ward	No. of unfilled posts?
Paediatricians?	*******		
Doctors? (MOs)			
Medical assistants? (COs)			
Nurses per dayshift? (NDs)			
Nurses during the nightshift? (NNs)			
Auxiliary staff per dayshift? (ADs)			
Auxiliary staff during the nightshift? (ANs)			

	Always	Mostly	Rarely	Never
Do you have electricity at your hospital?	☐	☐	☐	☐
Do you have back-up power supply in the case of a power cut (i.e. diesel generator)?	☐	☐	☐	☐
Do you have running water in the paediatric ward at your hospital?	☐	☐	☐	☐
Do you have soap and /or desinfectant for handwashing in the paediatric ward?	☐	☐	☐	☐
Are the patient washing and toilet facilities adequate?	☐	☐	☐	☐
Do children share beds with one-another?	☐	☐	☐	☐
Is transport available to send referrals to a specialist whenever it is needed?	☐	☐	☐	☐
**2. Laboratory support**				
**Are the following laboratory investigations available at your hospital:**	Always	Mostly	Rarely	Never
Blood glucose?	☐	☐	☐	☐
Haemoglobin?	☐	☐	☐	☐
Haematocrit?	☐	☐	☐	☐
Bilirubin	☐	☐	☐	☐
Microscopy or Rapid Diagnostic test (RDT)for malaria parasites?	☐	☐	☐	☐
CSF microscopy?	☐	☐	☐	☐
HIV-serology?	☐	☐	☐	☐
Are trained HIV counsellors available for pre- and post test counselling?	☐	☐	☐	☐
Bloodgrouping and crossmatch?	☐	☐	☐	☐

**4. Hospital supplies and equipment**					
**Is the following equipment available in your hospital:**		Always	Often	Rarely	Never
Oxygen?<br>Indicate source:	__ oxygen cylinder<br>__ oxygen concentrator<br>__ central supply	☐	☐	☐	☐
Flow-meters for oxygen?		☐	☐	☐	☐
Oxygen saturation monitor?		☐	☐	☐	☐
Equipment for the administration of oxygen?		☐	☐	☐	☐
Indicate which equipment you use:	_____ nasal prongs<br>_____ catheters<br>_____ masks?				
IV-fluids for paediatric use?		☐	☐	☐	☐
IV-giving sets with chambers for paediatric use?		☐	☐	☐	☐
Butterflies and/or cannulas of paediatric size?		☐	☐	☐	☐
NG-tubes, paediatric size?		☐	☐	☐	☐
Equipment for intra-osseous fluid administration?		☐	☐	☐	☐
Suction equipment?		☐	☐	☐	☐
Chest tubes?		☐	☐	☐	☐
Nebulisers for administration of salbutamol?		☐	☐	☐	☐
Indicate type of nebulizer:	___electricity driven<br>___oxygen driven<br>___footpump driven				
Spacers with masks for administration of metered doses (spray) of salbutamol?		☐	☐	☐	☐
Functional X-ray equipment?		☐	☐	☐	☐
Neonatal Ambu-bag and masks of different sizes (premature and full term size) for hand ventilation?		☐	☐	☐	☐
**Is the following equipment available in your hospital:**		Always	Often	Rarely	Never

Long term baby warming system / incubator	☐	☐	☐	☐
Photo-therapy equipment?	☐	☐	☐	☐
Scales for newborns?	☐	☐	☐	☐
Scales for children?	☐	☐	☐	☐
Otoscopes?	☐	☐	☐	☐
Thermometers?	☐	☐	☐	☐

**IV-fluids:**	Always	Often	Rarely	Never
Glucose 50%?	☐	☐	☐	☐
Glucose 10 %?	☐	☐	☐	☐
Glucose 5 %?	☐	☐	☐	☐
Normal saline?	☐	☐	☐	☐
Ringer's or Darrows or Hartmanns?	☐	☐	☐	☐
Half strength Ringers or Darrows?	☐	☐	☐	☐

**Antibiotics:**	Always	Often	Rarely	Never
Septrin	☐	☐	☐	☐
Ampicillin/ Amoxycillin? - Intravenous	☐	☐	☐	☐
- Oral	☐	☐	☐	☐
Benzyl Penicillin	☐	☐	☐	☐
Cloxacillin / Flucloxacillin? - Intravenous	☐	☐	☐	☐
- Oral	☐	☐	☐	☐
3rd generation Cephalosporins? (eg cefotaxime, ceftraixone)	☐	☐	☐	☐
Gentamicin (or Netilmicin)	☐	☐	☐	☐
Chloramphenicol - Intravenous	☐	☐	☐	☐
- Oral	☐	☐	☐	☐
Nalidixic acid	☐	☐	☐	☐
Ciprofloxacin / Norfloxacin / Ofloxacin	☐	☐	☐	☐

**Anti-Tb drugs:**	Always	Often	Rarely	Never
All anti-Tb drugs needed according to the national Tb control programme	☐	☐	☐	☐

**Anti-malaria drugs:**	Always	Often	Rarely	Never
Co-artem	☐	☐	☐	☐
Quinine for injection	☐	☐	☐	☐
Oral quinine	☐	☐	☐	☐
Amodiaquine - oral	☐	☐	☐	☐

List any other anti-malarial drugs used in the hospital?

1 ________________

2 ________________

**Anti-fungal drugs:**	Always	Often	Rarely	Never
Ketoconazole	☐	☐	☐	☐
Greseofulvin	☐	☐	☐	☐
Lamisil	☐	☐	☐	☐
Other, name	☐	☐	☐	☐

**Emergency and other drugs:**	Always	Often	Rarely	Never
Adrenaline for subcutaneous injection?	☐	☐	☐	☐
Prednisolone	☐	☐	☐	☐
Digoxin ?	☐	☐	☐	☐
Furosemide ?	☐	☐	☐	☐
Spironolactone?	☐	☐	☐	☐
Pethidine?	☐	☐	☐	☐
Ibuprofen (brufen)	☐	☐	☐	☐
Paracetamol	☐	☐	☐	☐
Mebendazole / Albendazole	☐	☐	☐	☐

**Anticonvulsants**	Always	Often	Rarely	Never
Diazepam or Paraldehyde for injection?	☐	☐	☐	☐
Phenobarbitone - Injection	☐	☐	☐	☐
- Oral	☐	☐	☐	☐

**Other drugs and formulations:**	Always	Often	Rarely	Never
Iron syrup	☐	☐	☐	☐
Iron tablets, _____ mg	☐	☐	☐	☐
Multivitamin preparation (oral)	☐	☐	☐	☐
Vitamin A oral	☐	☐	☐	☐
Vitamin K im injection	☐	☐	☐	☐
Special milk for malnourished children (F75 & F100)	☐	☐	☐	☐
Supplementary formula milk for neonates if EBM is inadequate.	☐	☐	☐	☐
ORS	☐	☐	☐	☐
Oral potassium supplement	☐	☐	☐	☐
Mineral mix for malnourished children	☐	☐	☐	☐
**Vaccines**	Always	Often	Rarely	Never
BCG vaccine	☐	☐	☐	☐
Measles vaccine	☐	☐	☐	☐
Polio vaccine	☐	☐	☐	☐
Pentavalent vaccine (DTP/Hib/HepB)	☐	☐	☐	☐

Healthworker Questionnaire

HN |	HW |	Date

Type of healthworker being interviewed	
Current place of work (ward, MCH etc)	
Are you **ever** in charge of the ward (eg at night) or the person responsible for the immediate medical management of new paediatric admissions?	Yes / No

Thinking about children admitted to this hospital do you think:

	Usually inadequate	Occ. inadequate	Satisfactory	Good quality
1 a) the accommodation (space / beds)	☐	☐	☐	☐
1 b) the toilets and washing facilities for patients	☐	☐	☐	☐
1 c) the cleanliness of the ward	☐	☐	☐	☐
1 d) the food given to the children is	☐	☐	☐	☐

We have asked you some specific questions about the children's ward. Are there other things about the hospital buildings / ward that you think are good or things that could be improved?

Date / /	ID | | . HW |

We now want to ask you about the people working on the ward and the facilities available.

	Usually inadequate	Occ. inadequate	Satisfactory	Plenty
2. Do you think the number of staff available to care for sick children are?	☐	☐	☐	☐
3. The availability of (the following) are:				
a) Drugs	☐	☐	☐	☐
b) Oxygen	☐	☐	☐	☐
c) Blood for transfusion	☐	☐	☐	☐
d) iv fluids	☐	☐	☐	☐
e) food / special milk for malnutrition	☐	☐	☐	☐
f) milk supplements for neonates	☐	☐	☐	☐
g) laboratory tests (eg. Hb)	☐	☐	☐	☐
4. What do you think of the time you have available to do your job in the best way you know how (as you were trained)?	☐	☐	☐	☐

Do you have problems with any other equipment or supplies that make it hard to look after sick children well or are supplies in general good?

Do you think the hospital lacks any important staff to help look after sick children or are the numbers and quality of staff in general good?

Date / /	ID | | . HW |

	Usually inadequate	Occ. inadequate	OK	Very good

5. If you have a problem with a sick child is the supervision / support (eg from more senior clinical staff) available to you?	☐	☐	☐	☐
If you have problems getting help when you think you need it is it because?<br><br>*..there are not enough skilled people to call?*<br><br>*..you are unable to contact the right people?*<br><br>*..the response to your request is too slow?*<br><br>*..another reason?*				
6. What do you think about the information / explanations families are given about their child's illness	☐	☐	☐	☐
7. Is the time you have to explain to the parents and children about their illness?	☐	☐	☐	☐

	Always	Often	Sometimes	Rarely	Never
8. Overall are you pleased with what this hospital is able to do to help sick children while they are in hospital?	☐	☐	☐	☐	☐

9. Are there any other things that you have not told us about that could be changed to improve the care of children in the hospital?

Caretaker Questionnaire

HN |	Age of interviewee:	Date	
Parent of observed case ID No.	| |	Date admitted:	
Relationship to patient			
Time on ward with child: <25% , 25-50% , 50-75% , > 75%			

I want to ask you some questions about (name of child)'s admission to hospital and what your feelings are about it – Do you have any thing to say now about the time your child was in hospital, good things or bad things that happened to you?

I now want to ask you about some specific aspects of your child's admission:

1) In particular I would like to know what you thought about the **time in outpatients**, before s/he was admitted:	Worse than expected	As expected	Better than expected
a) the amount of time you waited before being seen, *How long did you wait?*	☐	☐	☐
b) the politeness with which you were treated	☐	☐	☐
c) the care the doctor took over the examination (the completeness of his/her assessment of your child's problem)	☐	☐	☐

d) were you surprised that s/he was admitted?	Y / N	e) was the reason for admission explained to you?	Y / N

2. Were there any other good / bad things about the time your child spent in outpatients?

3. Once your child was on the ward what did you think about:	Worse than expected	As expected	Better than expected
a) the amount of space for you and your child to stay	☐	☐	☐
b) the place / bed where you and your child slept?<br><br>If worse what was bad about it?	☐	☐	☐
c) the place to wash and go to the toilet<br>*If worse what was bad about it?*	☐	☐	☐
d) the cleanliness of the ward	☐	☐	☐

Date / /	ID | | . HN | |

….are there other things about the ward itself or the hospital site that concern you?

3. Do you know what illness caused your child's admission?<br>*If yes what is the name of the illness?*					Y / N
a)Who gave you information about the illness? *(>1 choice is allowed)*	Doctors	Nurses	Other hosp. staff	No-one	Not appropriate
b)Who gave you information about the tests? *(>1 choice is allowed)*	Doctors	Nurses	Other hosp. staff	No-one	Not appropriate
c)Who gave you information about the treatment? *(>1 choice is allowed)*	Doctors	Nurses	Other hosp. staff	No-one	Not appropriate
4. Do you think the information you were given about your child's illness / treatment was?	Enough		Not enough		Not appropriate

If the information was not enough, what did you want to know more about?

5. Which people would you feel comfortable to put questions to about your child's illness, tests or treatment? *(>1 choice is allowed)*	Doctor	Nurses	Other hosp. staff	No-one	Not appropriate

Why did you not feel comfortable to put questions to the doctors / nurses / other staff?

We now want to ask you about what you thought about the staff looking after your child?

6. What was the attitude of the different types of staff towards you and your child most of the time	Polite, helpful	Rude, unhelpful	Acceptable
a) Doctors	☐	☐	☐
b) Nurses	☐	☐	☐
c) Cleaning / kitchen staff / subordinate staff	☐	☐	☐
c) Other hospital staff (eg nutritionists / Xray / physio etc)	☐	☐	☐

Date / /	ID | | . HN | |

7. What do you think of the condition of your child now (at the time of discharge)?	Very good	OK	Still poor
8. Do you think the amount of time they spent in hospital was?	Too long	Just right	Too short

9. Is your child to be sent home on medicines *(If no go to Q. 13)*	Y / N
10. About the medicines you should take home: Did the ward staff tell you how much to give? *(Please ask the caretaker to explain how they will dose)*	Y / N
11. Did the ward staff tell you how often to give the medicines to take home? *(Please ask the caretaker to explain how often they will give each medicine)*	Y / N
12. Did the ward staff tell you how long (the number of days) you should give the medicines for when you are at home? *(Please ask the caretaker to explain how long they will continue each medicine)*	Y / N

Please record any inconsistencies between the mothers reported understanding of discharge medication and the true dose, frequency and duration of discharge medication.

13. Years of primary education of interviewee	
14. Home residence (urban / rural)	
15. Was the interview conducted in a language in which the caretaker feels very comfortable (eg. their 'mother tongue')?	Y / N

Caretaker Questionnaire – Part 2 – Costs of Care.

	**Travel Information**	
1.	How long did it take to get here from your home (including the journey time and any waiting for transport)?	Minutes |___|___| Hours |___|___| ☐ Unknown
2.	What kind of transportation did you use to bring your child to this hospital or clinic?<br>*In case of multiple means of transportation during this trip, please tick only the transportation that was used for the longest distance).*	☐ Car = 1<br>☐ Bus / train = 2<br>☐ Matatu = 3<br>☐ Bicycle = 4<br>☐ Motorbike = 5<br>☐ Taxi = 6<br>☐ By foot = 7<br>☐ Boat = 8<br>☐ Ambulance = 9<br>☐ Other, specify: = 10<br>____________________
3.	If you paid for transportation to bring the child to the hospital or clinic, how much did you pay?	Ksh_________________<br>*(put **0** if no payment was made and 999 if don't know)*
4.	How many trips did you or other household members make to visit your child? *(Total numbers of round trips)*<br>*Examples:3 relatives' visit one time [**n = 3 trips**]*<br>*One relative visits three times [**n = 3 trips**]*	|___|___| (Put ***0*** *if no visit was made)*
5.	What kind of transportation did ***you*** use to come to this hospital or clinic to visit your child?<br>*(It concerns the last used transportation that has been used to visit your child).*<br>________________________	☐ Car = 1<br>☐ Bus / train = 2<br>☐ Matatu = 3<br>☐ Bicycle = 4<br>☐ Motorbike = 5<br>☐ Taxi = 6<br>☐ By foot = 7<br>☐ Boat = 8<br>☐ Other, specify = 9<br>______________________

6. If you paid for transportation, how much did ***you*** pay to visit this health care facility? *(Round trip, one person)*

If you used different means of transportation, please choose the one you used most often.

Ksh ________________

*(put **0** if no payment was made and 999 if don't know)*

Treatment Costs

15. Before visiting this facility, did you seek help from any of the following? How much did it cost you for Drugs, Tests, Consultation and other financial costs? **(Caregiver to list all the facilities visited, then ask the costs of each item for each place visited one at a time)**

Exp/Facil	Hospital	Priv clin	Pub clin	Pharm	Trad	Friend	Shop	Other
Drug								
Test								
Cons								
Othe cost								
Tot								

Key Exp = Expenditure, Facil = Facility, Priv clin = Private clinic, Pub clin = Public clinic, Pharm = Pharmacy, Trad = Traditional healer or herbalist, Othe cost = Other financial costs, Tot = Total costs.

16. How much did the household pay for: Drugs, Tests, Consultation, and Other fees for this visit or hospitalisation?

Item	Drug	Tests	Consultation Fee	Other fees	Total
Cost**(Put 0 if no payment and 999 if don't know)**					
Ability to pay	☐ Y ☐ N	☐ Y ☐ N	☐ Y ☐ N	☐ Y ☐ N	

17.	If you weren't here today, what would you be doing? (*Multiple responses allowed).*	☐ Nothing	=	1
		☐ Housework	=	2
		☐ Looking after my children	=	3
		☐ Working (specify)	=	4
		☐ Other (specify)	=	5
		☐ Don't know	=	6
18.	How much income have you or other family members lost as a result of taking care of your child instead of working?	Ksh ____________________________ *(put **0** if nothing and 999 if don't know)*		

Financing of the costs of treatment and transport

19. Has the illness affected the family financially?
☐ Yes
☐ No

20. Where did the money come from to pay for these expenses?

(Multiple responses allowed)

☐ Cutting down on other expenses = 1
☐ Using savings = 2
☐ Borrowing = 3
☐ Selling assets = 4
☐ Asking for donations from friends and relatives = 5
☐ Others, specify = 6

21. What is the total number of people in your household?

___________ Adults	__________ Children
___________18 –28 yrs	____________0-5 Yrs
_____________28-38 Yrs	_____________5-10 yrs
______________38-48 Yrs	_____________10-17 Yrs
________________48-58 Yrs	
________________58-68 Yrs	
_________________68+ Yrs	

22. What are the total expenses of the household where the child lives?

Item/ Amount	Per day	Per week	Per month
Food			
Education			
Rent			
Household items			
Medical/Health			
Total			

HEALTH WORKER MOTIVATION QUESTIONNAIRE

Hospital Code			Questionnaire No.			Date			
Health worker type			❑ MO	❑ CO		❑ Lab/Rad. Tech			
			❑ Pharmacist	❑ Nurse		❑ Other__________			
Health worker gender			❑ Male	❑ Female					
Area where you provide health services			❑ Paediatric ward	❑ Nursery / Maternity		❑ OPD Paed./MCH			
			❑ Lab/Rad	❑ Surgical/ Ortho ward		❑ Medical ward			
			❑ OPD Other	❑ Pharmacy					

Instructions

1. Please read the question carefully and answer. If you are unsure about anything, please ask one of the survey staff for explanation.
2. We would like you to think about your experience and motivation for working in this hospital. The information required is captured in the questions contained in this form.
3. In Section I, you will find basic questions on your training and working experience to date. Please tick the appropriate box or fill in the box.

*3a. In the case of **Medical** and **Clinical Officers**, please provide the clinician code and proceed to Section II.*

*3b. For **all other cadres**, please complete questions 2-4 in section I and proceed to section II.*

4. In Section II, each question has five possible answers. To answer any question, please tick the box you that represents your answer like this:

	Strongly agree	*Agree*	Neutral	Disagree	Strongly disagree
My job requires a variety of knowledge and skills	❑ ✓	❑	❑	❑	❑

SECTION 1:

Clinician code (*for Medical and Clinical Officers only*) **[___|___]**

<table>
<tr><th colspan="4">Training</th></tr>
<tr><td colspan="2">What is your basic training level and what year did you complete basic training?</td><td>*{Circle one answer}*

1=Clinical Officer (*CO)*
2=Medical officer *(MBChB)*
3=Enrolled Nurse *(KEN/KECHN)*
4=Registered Nurse *(KRN/KRCHN)*
5=Other______________(specify)</td><td>Year of completion
|__|__|__|__|</td></tr>
<tr><td colspan="2">For Nurses, have you undertaken any upgrading course?</td><td>*{Circle one answer}*

Y=Yes

N=No</td><td></td></tr>
<tr><td>Have you undergone any major post basic training?</td><td>*{Circle one answer}*

Y=Yes

N=No</td><td>**If <u>YES</u>, which one?**

{Circle one answer}

1=Advanced diploma
2=MMed(Paediatrics)
3=MMed(Other)
4=MPH
5=Other__________(specify)</td><td>Year of completion
|__|__|__|__|</td></tr>
<tr><td colspan="2">Please summarise where you have <u>MAINLY</u> worked since you graduated and how many months you spent in each place?</td><td colspan="2"><table><tr><th>Facility</th><th>Years</th><th>Months</th></tr><tr><td>1.National hospital</td><td></td><td></td></tr><tr><td>2.Provincial hospital</td><td></td><td></td></tr><tr><td>3.District hospital</td><td></td><td></td></tr><tr><td>4.Health center</td><td></td><td></td></tr><tr><td>5.Dispensary</td><td></td><td></td></tr><tr><td>6.Mission hospital</td><td></td><td></td></tr><tr><td>7.Private hospital</td><td></td><td></td></tr><tr><td>8.Research institute</td><td></td><td></td></tr><tr><td>9.Other_______(specify)</td><td></td><td></td></tr></table></td></tr>
<tr><td colspan="2">For **ALL**, did you fill in **THIS** questionnaire during the first survey (July-August 2006)</td><td colspan="2">*{Circle one answer}*

Y=Yes

N=No</td></tr>
</table>

SECTION II					
	Strongly agree	*Agree*	Neutral	Disagree	Strongly disagree
I am punctual about coming to work	❑	❑	❑	❑	❑
I am glad that I work for this facility rather than other facilities in the country	❑	❑	❑	❑	❑
Overall, I am very satisfied with my job	❑	❑	❑	❑	❑
I always complete my tasks efficiently and correctly	❑	❑	❑	❑	❑
This job makes me feel good about myself	❑	❑	❑	❑	❑
These days, I feel motivated to work as hard as I can	❑	❑	❑	❑	❑
This hospital really inspires me to do my very best on the job	❑	❑	❑	❑	❑
I am satisfied with the opportunity to use my abilities in my job	❑	❑	❑	❑	❑
I am a hard worker	❑	❑	❑	❑	❑
I am proud to be working for this hospital	❑	❑	❑	❑	❑

Thank You.
